# Structural Immunology of SARS‐CoV‐2

**DOI:** 10.1111/imr.13431

**Published:** 2024-12-27

**Authors:** Meng Yuan, Ian A. Wilson

**Affiliations:** ^1^ Department of Integrative Structural and Computational Biology The Scripps Research Institute La Jolla California USA; ^2^ The Skaggs Institute for Chemical Biology The Scripps Research Institute La Jolla California USA

**Keywords:** COVID‐19, neutralizing antibody, SARS‐CoV‐2, spike, vaccine, viral evolution

## Abstract

The SARS‐CoV‐2 spike (S) protein has undergone significant evolution, enhancing both receptor binding and immune evasion. In this review, we summarize ongoing efforts to develop antibodies targeting various epitopes of the S protein, focusing on their neutralization potency, breadth, and escape mechanisms. Antibodies targeting the receptor‐binding site (RBS) typically exhibit high neutralizing potency but are frequently evaded by mutations in SARS‐CoV‐2 variants. In contrast, antibodies targeting conserved regions, such as the S2 stem helix and fusion peptide, exhibit broader reactivity but generally lower neutralization potency. However, several broadly neutralizing antibodies have demonstrated exceptional efficacy against emerging variants, including the latest omicron subvariants, underscoring the potential of targeting vulnerable sites such as RBS‐A and RBS‐D/CR3022. We also highlight public classes of antibodies targeting different sites on the S protein. The vulnerable sites targeted by public antibodies present opportunities for germline‐targeting vaccine strategies. Overall, developing escape‐resistant, potent antibodies and broadly effective vaccines remains crucial for combating future variants. This review emphasizes the importance of identifying key epitopes and utilizing antibody affinity maturation to inform future therapeutic and vaccine design.

The Coronavirus disease 2019 (COVID‐19) pandemic, caused by severe acute respiratory syndrome coronavirus 2 (SARS‐CoV‐2), has resulted in over 776 million infections and seven million deaths as of October 2024 [1]. Although vaccines and therapeutic antibodies have provided significant protection for public health, the continuous evolution of SARS‐CoV‐2, particularly mutations in the spike (S) protein, has enabled the virus to evade many of these defenses. In this review, we focus on the key sites on the SARS‐CoV‐2 S protein targeted by neutralizing antibodies (nAbs) and evaluate their susceptibility to escape mutations. We also review public antibodies targeting different regions of the SARS‐CoV‐2 S protein and discuss the effects of antibody allelic polymorphisms on the recognition of pathogenic antigens. Furthermore, we discuss strategies for developing the next generation of broadly potent neutralizing antibodies against SARS‐CoV‐2.

## 1 The S Protein Is a Major Target for Anti‐SARS‐CoV‐2 Antibodies and Vaccines

**FIGURE 1:**
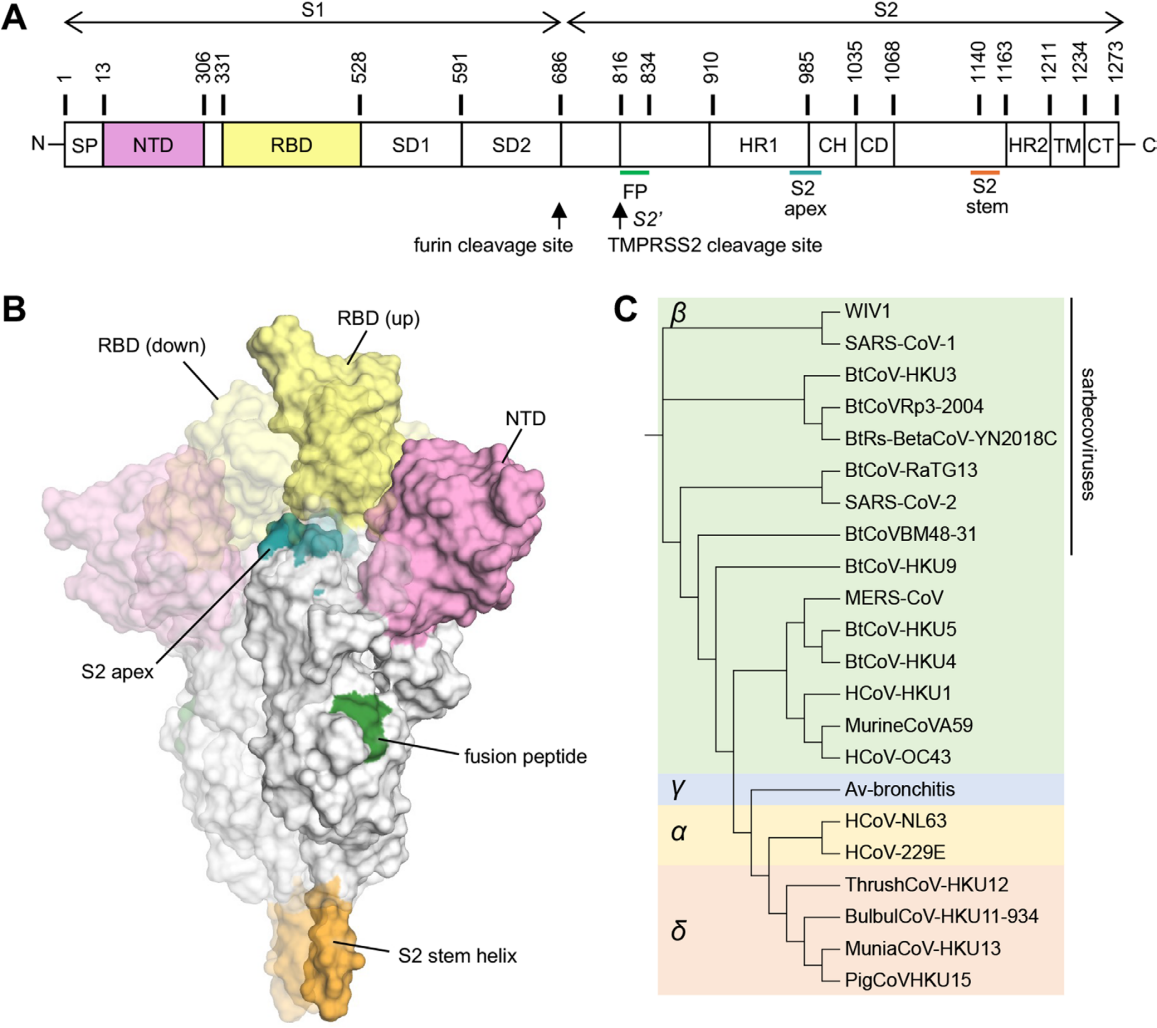
Overall structure of the SARS‐CoV‐2 spike (S) protein. (A) Schematic representation of the SARS‐CoV‐2 S protein. (B) A prefusion state of the SARS‐CoV‐2 S protein with one RBD in the “up” state (PDB 7N1Q [2]). The other two protomers with RBDs in “down” state are represented by a more faded surface. The color scheme of the highlighted regions is the same as in panel A. (C) Phylogenetic tree of coronaviruses. The phylogenetic tree was calculated by Clustal Omega [3] and edited by Interactive Tree Of Life (iTOL) [4].

SARS‐CoV‐2 utilizes its spike or S protein to engage human angiotensin‐converting enzyme 2 (ACE2) as an initial step in viral entry. The S protein serves as a primary target of the human immune response and is the focus of most therapeutic antibodies and vaccines. The S protein is a metastable, trimeric, glycosylated fusion protein composed of two subunits, S1 and S2 (Figure 1A,B). The S1 subunit comprised a signal sequence (SP), N‐terminal domain (NTD), receptor‐binding domain (RBD), subdomain 1 (SD1), and subdomain 2 (SD2). The S2 subunit contains the fusion peptide (FP), heptad repeat 1 (HR1), central helix (CH), connector domain (CD), heptad repeat 2 (HR2), transmembrane (TM) domain, and cytoplasmic tail (CT). The interaction between the RBD in the prefusion S and ACE2 initiates cell entry. The RBD shifts between “up” and “down” states with the receptor‐binding site (RBS) hidden in the “down” state. The RBD becomes accessible to ACE2 when in the “up” state. The interaction of ACE2 triggers a transition from the prefusion to postfusion state of S emanating from a large conformational change in HR1, which exposes the fusion peptide and mediates membrane fusion with the target cells.

The S protein is major target for nAbs against SARS‐CoV‐2. Several vaccines, such as mRNA and adenoviral vector vaccines, encode or present the S protein as the sole SARS‐CoV‐2 antigen to elicit an immune response. Numerous studies have identified potent nAbs that bind to various epitopes on the S protein, particularly within the RBS on the S1 subunit [5, 6]. These antibodies can prevent the interaction between the viral S protein and the host cell receptor ACE2, thereby inhibiting cell entry. Additionally, some nAbs target conserved epitopes, offering potential broad‐spectrum protection against SARS‐CoV‐2 variants, sarbecoviruses, betacoronaviruses, or even coronaviruses in other genera [7, 8, 9, 10]. The development of therapeutic antibodies and vaccines has largely focused on eliciting robust nAb responses against the S protein to confer protection against COVID‐19. We previously assembled a dataset of 8048 human monoclonal antibodies (mAbs) against SARS‐CoV‐2 from over 200 COVID‐19 patients and vaccinees [11], where 7974 of the mAbs target the S protein, including 5002 that bind to RBD, 513 to NTD, and 890 to the S2 subunit.

## 2 Multiple Coronaviruses Have Caused Human Disease

Coronaviruses (CoVs) are named for their crown‐like appearance, with spike proteins protruding from the viral surface. CoVs are enveloped viruses containing single‐stranded, positive‐sense RNA (+ssRNA) genomes, ranging from approximately 26 to 32 kilobases (kb). Coronaviruses are a family of viruses comprising four genera: *Alphacoronavirus*, *Betacoronavirus*, *Gammacoronavirus*, and *Deltacoronavirus* (Figure 1C). In addition to SARS‐CoV‐2, several other coronaviruses are capable of human‐to‐human transmission and have caused severe human disease. SARS‐CoV‐1, another member of the sarbecovirus lineage within betacoronaviruses, also utilizes human ACE2 as its host cell receptor. This virus resulted in the severe acute respiratory syndrome (SARS) outbreak in 2002–2003, resulting in over 8000 cases and 774 deaths [12]. MERS‐CoV, another betacoronavirus, can cause Middle East Respiratory Syndrome (MERS), a severe viral respiratory illness. MERS‐CoV was first identified in Saudi Arabia in 2012, and approximately 35% of MERS cases reported to WHO have resulted in death. MERS‐CoV primarily targets dipeptidyl peptidase 4 (DPP4) as its major human receptor. Additionally, seasonal coronaviruses, which cause the common cold with mild to moderate upper respiratory symptoms, include HCoV‐HKU1 and HCoV‐OC43 (betacoronaviruses), as well as HCoV‐NL63 and HCoV‐229E (alphacoronaviruses).

## 3 Evolution of SARS‐CoV‐2 S Exhibits Antibody Escape and Stronger Receptor Binding

**FIGURE 2:**
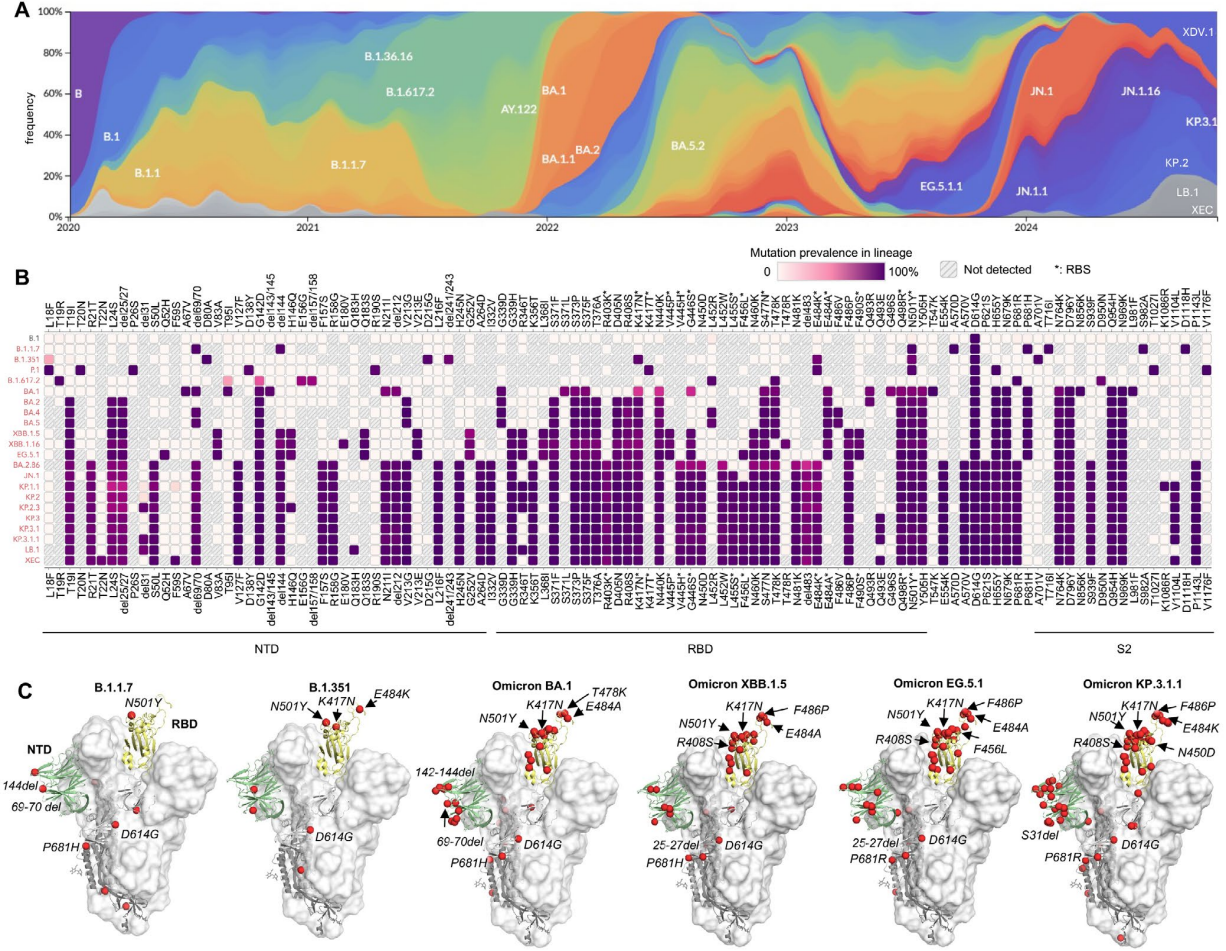
Evolution of the SARS‐CoV‐2 S protein. (A) Frequencies of SARS‐CoV‐2 variants since the beginning of the COVID‐19 pandemic (colored by Pangolin Pango Lineage [13]) as calculated by Nextstrain [14] using data from GISAID [15]. (B) Prevalent mutations of SARS‐CoV‐2 variants as calculated by Outbreak.info [16]. Mutations with > 75% prevalence are shown here. RBS residues (defined as buried surface area [BSA] > 0 Å^2^ as calculated by PISA [Proteins, Interfaces, Structures and Assemblies]) [17] are highlighted with asterisks. (C) Increasing number of mutations on the SARS‐CoV‐2 S protein during virus evolution. Mutated residues are represented by red spheres.

The emergence of many SARS‐CoV‐2 variants, particularly the omicron variant and subvariants that emerged in November 2021 (Figure 2A), have largely increased transmissibility and reduced immune protection induced by previous infections or vaccinations. The latest subvariant as of October 2024, known as KP.3.1.1, has accumulated 61 mutated amino acids on the S protein compared to the original Wuhan strain, with 29 of these on the RBD (Figure 2B,C).

Only a few months after the initial discovery of COVID‐19, a lineage of SARS‐CoV‐2 carrying a D614G mutation in the S protein emerged and rapidly spread worldwide [18]. D614G shifts the S protein conformation toward an ACE2‐binding‐prone state and enhances viral fitness [19, 20]. On the basis of the conformational stability conferred by D614G [21], eight to eleven more mutations and deletions on the S protein emerged in the alpha (B.1.1.7), beta (B.1.351), gamma (P.1), and delta (B.1.617.2) variants, which reduced vaccine efficacy and sera neutralization [22]. The human immune response against SARS‐CoV‐2 was largely primed and imprinted by prior infection of early variants or vaccines designed based on the prototype SARS‐CoV‐2. Viral evolution has since followed trajectories in which emerging variants exhibit immune escape while maintaining or enhancing viral fitness.

### 3.1 Mutations on the S Protein Enable Antibody Escape

**FIGURE 3:**
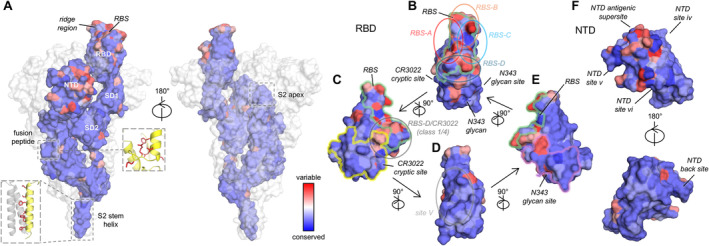
Antibody sites and conservation of the SARS‐CoV‐2 S protein. Sequence conservation of SARS‐CoV‐2 variants are mapped on a prefusion S spike and its domains (PDB 7N1Q [2]) by ConSurf [23]. The color coding is consistent throughout this figure. (A) Sequence conservation of SARS‐CoV‐2 variants plotted on a SARS‐CoV‐2 S structure. B‐cell epitopes on S2 stem and fusion peptide are usually cryptic in the prefusion state and the epitope residues highlighted as red sticks in the boxes on the side. (B–E) Antibodies targeting different epitopes on the receptor‐binding domain (RBD) are classified as RBS‐A, ‐B, ‐C, ‐D, CR3022 cryptic site, N343 glycan site, and site V, which are indicated in the panels that present different views of the RBD. (F) Antibodies targeting different epitopes on the N‐terminal domain (NTD).

nAbs targeting the RBS usually exhibit higher potency compared to non‐RBS antibodies, likely because they directly block receptor binding. The RBS has been a heavily mutated region of the S protein of SARS‐CoV‐2 during its evolution (Figure 3), leading to significant antibody escape. Mutations at positions K417 and E484, located in major sites targeted by potent nAbs, emerged in beta (B.1.351) and gamma (P.1) variants and facilitated escape from major classes of nAbs targeting the RBS [24]. Strikingly, the founding omicron variant BA.1 (B.1.1.529) displayed numerous mutations in the S protein (including K417 and E484 mutations; Figure 2B,C), resulting in substantial immune evasion [22, 25, 26, 27, 28, 29, 30]. Omicron rapidly spread worldwide at an unprecedented pace, becoming the dominant variant within 2 months (Figure 2A). Since then, omicron subvariants have continued to dominate SARS‐CoV‐2 transmission, with additional mutations enabling even greater evasion of the preexisting human immune response. For example, instead of a S371L/S373P/S375F trio of mutations that emerged in omicron BA.1, BA.2 replaced L371 with F371, while the other two mutations at 373 and 375 remained the same as BA.1. This F371 mutation resulted in a further clash with the glycan at the N343 glycosylation site, reducing neutralization by broadly neutralizing antibody (bnAb) S309 [31, 32]. LY‐CoV1404 is another therapeutic nAb that neutralized a broad spectrum of SARS‐CoV‐2 variants with extraordinary potency; however, mutations K444N/T, V445P/H, and G446S, which emerged in newer variants such as BQ.1.1 and XBB, allowed escape from LY‐CoV1404. AZD8895 (Tixagevimab/COV2‐2196) targets the ridge region of the RBS, centered around a hydrophobic F486 at the tip of the ridge with which its forms extensive interactions. F486 is mutated to S, V, or P in later variants, leading to antibody escape from AZD8895 and many other antibodies targeting this region.

### 3.2 Mutations on the S Protein Enhance Binding With ACE2

Binding between the RBD of the SARS‐CoV‐2 S protein to the host receptor ACE2 is the initial step of viral entry. Surface plasmon resonance (SPR) measurements have demonstrated that newer variants exhibit stronger binding to the receptor [33]. The dissociation constant (*K*_D_) between the human ACE2 and SARS‐CoV‐2 D614G S trimer was reported to be 152 nM, decreasing to 73 nM for XBB.1.5 and 92 nM for EG.5. The stronger binding to ACE2 of the S proteins of the more recent SARS‐CoV‐2 variants compared to D614G is due to a slower dissociation rate, which compensates for a reduction in association rates [33].

During SARS‐CoV‐2 evolution, many mutated residues have contributed to stronger binding to the human receptor. The N501Y mutation forms an additional π–π interaction with ACE2‐Y41 and a π–cation interaction with K353, strengthening S/ACE2 binding by fourfold [34]. F486 interacts with a hydrophobic region of ACE2, comprising L79, M82, and Y83. F486V (BA.4/5, BQ.1, and BQ.1.1) and F486S (XBB.1), which reduced binding to ACE2 [35, 36], while a P486 mutation that emerged in XBB.1.5 and later variants contributed to stronger binding affinity to ACE2 compared to S486 [37, 38]. The ACE2 binding to a P486 spike did not substantially differ from the ancestral F486 [36, 39]. In addition to single mutations that result in stronger ACE2 binding, some mutations on the S protein have demonstrated an epistatic effect than can rescue binding of a deleterious mutation. For example, K417N emerged in beta and omicron subvariants. K417N alone reduces binding between the RBD to ACE2 by approximately sixfold. However, the reduced affinity is compensated by N501Y, which is also present in these variants [24]. Likewise, Q498R emerged in omicron and subvariants when N501Y is present. Although Q498R alone weakly reduces ACE2 affinity in the Wuhan RBD, N501Y facilitates a large enhancement in affinity when combining with Q498R, in a synergistic manner [40]. Additionally, Q493 emerged in the KP.3 lineage and is directly involved in ACE2 binding. The recent Q493E single mutation would ordinarily reduce S/ACE2 binding; however, in the context of KP.3, another mutation, F456L, provided an epistatic effect, largely enhancing binding with ACE2 [41, 42]. Overall, stronger binding of the S protein to the host receptor may contribute to the increased transmissibility of newer SARS‐CoV‐2 variants.

## 4 A Comprehensive Overview of nAb Sites on the SARS‐CoV‐2 S Protein

The S protein of SARS‐CoV‐2 comprised RBD, NTD, and the S2 subunit (Figure 3). The RBD is a major target for nAbs [43]. This immunodominant domain is the target of 90% of the neutralizing activity present in SARS‐CoV‐2 immune sera [44]. Among the 8048 human antibodies that we previously extracted and evaluated from 88 research publications and 13 patents, 5002 bind to RBD, 513 to NTD, and 890 to the S2 subunit [11]. Depletion of RBD‐binding antibodies from the plasma of COVID‐19 patients and vaccinees result in a significant loss of neutralization activity [45]. However, the RBS is the most variable region of the SARS‐CoV‐2 S protein. While many highly potent nAbs target this site, mutations frequently enable the virus to evade these antibodies, while still retaining or even gaining in affinity to ACE2. Some NTD‐targeting antibodies also exhibit high neutralization potency against SARS‐CoV‐2 [46, 47].

We have classified the antibody‐targeting regions on the SARS‐CoV‐2 RBD into seven distinct sites, four RBS sites (RBS‐A, B, C, and D), CR3022 cryptic site, N343 glycan site, and site V (Figure 3B–E) [32, 48]. As shown in the top view of the RBD (Figure 3B), RBS‐A, B, C, and D are located on the left, front top, right, and front bottom of the RBS, respectively. The CR3022 cryptic and N343 glycan sites are at the lower left and lower right areas of the RBD, respectively (Figure 3B), while site V is on the flank of the RBD (Figure 3D). The RBS is the major region targeted by nAbs and bind to the different subareas with various angles of approach to the RBD. Unlike most viruses that engage with host receptors through relatively conserved receptor‐binding sites, the RBS of SARS‐CoV‐2 is the most variable region within the S protein (Figure 3). These mutations induce significant immune escape from wild‐type vaccines, where many of the elicited nAbs, and almost all FDA‐approved therapeutic antibodies, are rendered ineffective by at least one major SARS‐CoV‐2 variant.

### 4.1 S1 Domain

#### 4.1.1 RBS‐A

RBS‐A is located on the left side of the RBS (Figure 3B) and largely overlaps with the RBS. Most RBS‐A antibodies bind only to RBDs in the “up” conformation, as they would clash with the adjacent protomer in the S trimer if bound to an RBD in the “down” conformation [49, 50]. However, in rare cases, RBS‐A antibodies can also bind to a down‐RBD by inducing a conformational change in the adjacent protomer [51]. RBS‐A antibodies correspond to the “class I” antibodies classified by Barnes et al. [52].

##### 4.1.1.1 Public Antibodies Targeting RBS‐A

**FIGURE 4:**
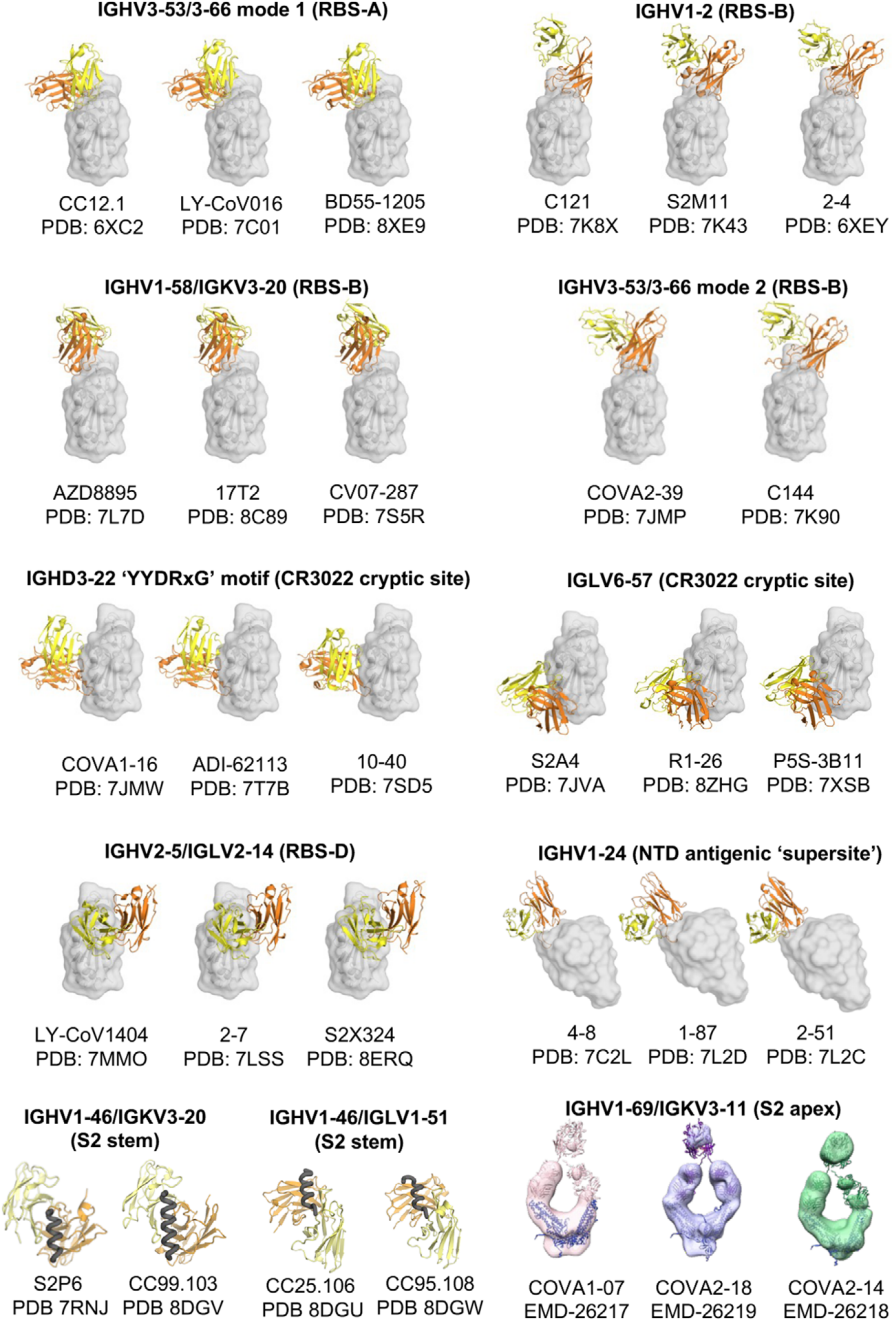
Public classes of antibodies targeting the SARS‐CoV‐2 S protein. Antibody/antigen complex structures are from the Protein Data Bank (PDB) with the heavy and light chains of the antibodies shown in orange and yellow, respectively. The antigens are shown in transparent gray for RBD and NTD, and as black tubes for the S2 stem helices. The images of the negative stain EM maps of the anti‐S2‐apex antibodies are from the Electron Microscopy Data Bank (EMDB). High‐resolution structures of these antibodies are not available.

Many RBS‐A antibodies are encoded by IGHV3‐53/3‐66 germline genes, which are highly enriched in COVID‐19 patients and vaccinees (Table 1 and Figure 4) [11, 49, 56]. These public antibodies are usually highly potent, with low somatic hypermutation (SHM), and structurally convergent. We previously determined high‐resolution crystal structures of IGHV3‐53/3‐66 antibodies, revealing the structural characteristics of these antibodies in recognizing the SARS‐CoV‐2 RBD. The germline “NY” and “SGGS” motifs in CDRs H1 and H2, respectively, extensively interact with the antigen, and the short CDR H3 (H3 ≤ 11 amino acids) are accommodated in a relatively flat region of the RBD [49]. IGHV3‐53/3‐66 antibodies targeting the SARS‐CoV‐2 S protein often contain convergent SHMs, such as Y58F, F27V, T28I, and S31R, which exhibit more favorable interactions with the RBD and improved affinity [11, 56, 74, 75, 76]. Some approved therapeutic antibodies targeting the RBS‐A site are also encoded by IGHV3‐53/3‐66 germline genes, including LY‐CoV016 (i.e., CB6, JS016, Etesevimab) [53], P2C‐1F11 (i.e., BRII‐196, Amubarvimab) [77], and BD‐604 (DXP‐604) [78]. Many IGHV3‐53/3‐66 antibodies targeting RBS‐A are sensitive to the K417N/T mutation at the center of the RBS‐A site, including variants beta (K417N), gamma (K417T), and omicron subvariants (K417N) [49]. For example, CC12.1, CC12.3, COVA2‐04, and LY‐CoV016 are evaded by K417N‐containing variants [24, 26]. Notably, however, some IGHV3‐53/3‐66 antibodies have been found to be insensitive to K417N/T mutations, and are able to retain their activity against beta, gamma, and omicron variants [51, 79, 80, 81, 82, 83]. Mutations at L455 and F456 further reduce the activity of IGHV3‐53/3‐66 antibodies [56].

**TABLE 1:** Public classes of antibodies targeting SARS‐CoV‐2 S protein.

Site	Germline	Representative mAbs	Variants neutralized	References
RBS‐A	IGHV3‐53/3‐66 (mode 1^a^)	CC12.1	Escaped by beta, gamma, and omicron subvariants	[24, 49]
		LY‐CoV016	Escaped by beta, gamma, and omicron subvariants	[53, 54]
		BD55‐1205	All SARS‐CoV‐2 VOCs as of October 2024	[36, 55]
RBS‐B	IGHV1‐2	C121	Escaped by beta, gamma, and omicron subvariants	[52]
		2–4	Escaped by beta, gamma, and omicron subvariants	[56]
		S2M11	Escaped by beta, gamma, and omicron subvariants	[46]
	IGHV1‐58/IGKV3‐20	AZD8895	Largely reduced by omicron subvariants	[57]
		17T2	Largely reduced by XBB.1.5 and later variants	[58]
	IGHV3‐53/3‐66 (mode 2^a^)	C144	Escaped by beta, gamma, and omicron subvariants	[52]
		COVA2‐39	Escaped by beta, gamma, and omicron subvariants	[59, 60]
RBS‐D	IGHV2‐5/IGLV2‐14	LY‐CoV1404	Cross‐react with sarbecovirus clade 1b Escaped by BQ.1, XBB, and later variants	[61, 62, 63, 64]
		S2X324	Cross‐react with sarbecovirus clade 1b Escaped by BQ.1, XBB, and later variants	[61, 63, 64, 65]
CR3022 cryptic site	IGHD3‐22 “YYDxxG” motif	COVA1‐16	Cross‐react with sarbecoviruses and SARS‐CoV‐2 variants including omicron subvariants	[59, 66]
		ADI‐62113	Cross‐react with sarbecoviruses and SARS‐CoV‐2 variants including omicron subvariants	[67]
		10–40	Cross‐react with sarbecoviruses and SARS‐CoV‐2 variants including omicron subvariants	[68]
NTD antigenic supersite	IGHV1‐24	4A8	Escaped by alpha and later variants	[46, 69]
		TXG‐0078	Cross‐react with 229E, OC43, HKU1 and SARS‐CoV‐2 variants beta, gamma, and kappa	[70]
S2 apex	IGHV1‐69/IGKV3‐11	COVA1‐07	Bind to a region highly conserved across coronaviruses, weakened by delta and omicron subvariants	[71, 72]
		COV2‐14	Bind to a region highly conserved across coronaviruses, weakened by delta and omicron subvariants	[71, 72]
S2 stem helix	IGHV1‐46/IGKV3‐20	S2P6	Cross‐react with betacoronaviruses and SARS‐CoV‐2 variants including omicron subvariants	[10]
		CC99.103	Cross‐react with betacoronaviruses and SARS‐CoV‐2 variants including omicron subvariants	[7]
		COV89‐22	Cross‐react with betacoronaviruses and SARS‐CoV‐2 variants including omicron subvariants	[73]
	IGHV1‐46/IGLV1‐51	CC25.106	Cross‐react with betacoronaviruses and SARS‐CoV‐2 variants including omicron subvariants	[7]
		CC95.108	Cross‐react with betacoronaviruses and SARS‐CoV‐2 variants including omicron subvariants	[7]

^a^ IGHV3‐53/3‐66 binding mode 1 is usually bound by antibodies with short CDR H3 (length ≤ 11 amino acids, Kabat numbering), while binding mode 2 by antibodies with a longer and more normal length CDR H3 [49, 60].

CS23 is an IGHV3‐53‐encoded antibody that binds to the RBD of the SARS‐CoV‐2 beta variant [83]. The antibody was isolated from an unvaccinated convalescent with a de novo beta infection. Interestingly, CS23 is specific to variants that carry the K417N/T mutations, such as beta, gamma, and omicron, whereas its affinity to K417‐RBD was markedly reduced. We previously determined a crystal structure of the beta RBD in complex with CS23 [83]. CS23 still engages the RBD through the canonical IGHV3‐53/3‐66 binding pose (mode 1), but has a unique ST turn in its CDR H3 and directs V_H_ M98 toward the pocket N417. Compared to K417, the shorter N417 flips inward and vacates its original pocket allowing interaction with V_H_ M98 and thereby exhibiting a N417‐specific interaction. Despite being largely evaded by SARS‐CoV‐2 mutations, a number of IGHV3‐53/3‐66 antibodies have retained activity during SARS‐CoV‐2 evolution. For example, IGHV3‐53/IGKV3‐20 antibodies CAB‐A17 and CAB‐A18, isolated from COVID‐19 convalescents who were infected in early 2020, exhibited high neutralization potency against SARS‐CoV‐2, with minimal reduction against omicron [84]. Some IGHV3‐53/IGKV1‐9 antibodies also retained high potency to omicron variants BA.1 and BA.2 (e.g., R40‐1C8 [51, 85, 86]). Cv2.3194 strongly neutralizes omicron BA.2‐ and BA.4/5‐derived subvariants, but lost activity against EG.5 and JN.1 [87]. Another study showed that preexisting B‐cell memory of IGHV3‐53/3‐66 antibodies was recalled following omicron breakthrough infection [81]. Some of the recalled IGHV3‐53/3‐66 antibodies exhibited potent cross‐neutralization and cross‐reactivity to later omicron variants. Moreover, IGHV3‐53/3‐66 memory B cells from breakthrough infections have been shown to undergo somatic hypermutation and affinity‐dependent selection, which broadened their specificity to recent variants, such as BA.5 and KP.3 [88, 89]. ZCP3B4 and ZCP4C9 are RBS‐A‐targeting IGHV3‐53/3‐66 nAbs isolated from COVID‐19 convalescents after omicron BA.2/BA.5 breakthrough infection. They neutralize a broad spectrum of SARS‐CoV‐2 variants including the currently circulating JN.1 and KP.2 [76]. BD55‐1205, an IGHV3‐53/3‐66 nAb that targets RBS‐A, has maintained strong neutralization activity against all major circulating SARS‐CoV‐2 variants as of October 2024. This highly enriched public class of antibodies, despite mostly being evaded, have demonstrated potential to maintain breadth and potency in some of these types of antibodies, providing a promising germline‐targeting strategy for vaccine design.

In addition to IGHV3‐53/3‐66 antibodies, nAbs encoded by other germlines have also been reported to target RBS‐A. For example, S2K146 exhibited strong neutralization against SARS‐CoV‐2 and a broad spectrum of variants, including omicron variants BA.2 and EG.5.1, but was evaded by BA.2.86 and JN.1 [90, 91]. S2K146 is encoded by IGHV3‐43 and IGLV1‐44. AZD3152, another non‐IGHV3‐53/3‐66 antibody targeting RBS‐A, remained effective up to the JN.1 variant, although it showed reduced potency against later variants containing the F456L mutation [92]. AZD3152 was affinity matured in vitro from its parental mAb Omi‐42 [93], which is encoded by IGHV3‐9 and IGLV2‐8. Another bnAb, VIR‐7229 (closest matches to IGHV3‐30 and IGLV2‐11 as predicted by IgBLAST [94]), was affinity matured from S2V29, isolated from a vaccinated COVID‐19 convalescent. VIR‐7229 exhibited extraordinary neutralizing potency and breadth, including to recent SARS‐CoV‐2 variants EG.5, BA.2.86, and JN.1, as well as other sarbecoviruses, and also targets RBS‐A. These antibodies bind to the RBD through slightly different epitopes compared to the canonical IGHV3‐53/3‐66 antibodies (Figure S1). Overall, despite the high variability of RBS‐A, we demonstrate that it is a highly promising target for eliciting broad and potent nAbs and for vaccine design, suitable for both germline‐targeting and nongermline‐targeting strategies.

#### 4.1.2 RBS‐B

RBS‐B is located at the middle front of the RBS (Figure 3B). Most RBS‐B antibodies can bind to RBDs in both “up” and “down” conformations. RBS‐B antibodies are categorized within “class 2” as classified by Barnes et al. [52] Many RBS‐B antibodies are highly potent. For example, therapeutic antibodies REGN10933 (Casirivimab), AZD8895 (Tixagevimab, COV2‐2196), CT‐P59 (Regdanvimab), and LY‐CoV555 (Bamlanivimab, LY3819253) target RBS‐B and potently neutralize the ancestral SARS‐CoV‐2. RBS‐B antibodies are encoded by various germline genes, including IGHV1‐2, IGHV1‐58, IGHV1‐69, and IGHV3‐53/3‐66 (usually with long CDRs H3), which encode structurally convergent or public antibodies, as well as other germline genes. RBS‐B is highly variable (Figure 3B), with E484 located in this site. Recognition of E484 is crucial for many antibodies targeting RBS‐B. Mutations at E484 in beta, gamma, and omicron subvariants substantially reduce the neutralization potency of RBS‐B‐targeting antibodies, as well as the neutralizing capacity of human convalescent and postvaccination sera [24, 32, 95, 96].

##### 4.1.2.1 Public Antibodies Targeting RBS‐B

Most IGHV3‐53/3‐66 antibodies have a short CDR H3 (≤ 11 amino acids) and target the RBS‐A epitope, while some IGHV3‐53/3‐66 antibodies with longer CDRs H3 (≥ 15 amino acids) cannot be accommodated by the canonical binding mode (mode 1; Figure 4) [49, 60, 74]. These antibodies (e.g., COVA2‐39, C144) are also structurally convergent, utilizing mode 2. They target a different epitope on the RBD, RBS‐B (Table 1 and Figure 4). Mode‐2 IGHV3‐53/3‐66 antibodies are sensitive to mutations at RBD‐E484, which is mutated in variants such as beta (E484K), gamma (E484K), and omicron (E484A, E484K).

IGHV1‐2 is another frequently used germline gene encoding anti‐SARS‐CoV‐2 RBD antibodies. Some IGHV1‐2 antibodies bind to the RBS‐B epitope through a similar binding mode (Table 1 and Figure 4) [24, 97]. IGHV1‐2 antibodies in this binding mode are also sensitive to E484 mutations, where a single mutation E484K leads to complete escape from binding and neutralization by IGHV1‐2 antibodies (e.g., C121, 2–15) [24, 32, 52, 96]. The beta variant contains the E484K mutation. SARS‐CoV‐2‐targeting IGHV1‐2 antibodies were rarely found in patients infected with the beta variant [83], further confirming the sensitivity of IGHV1‐2 antibodies to E484 mutations. The V_H_^26^GYTFTG^31^, ^50^WIXPXSGT^57^, and ^73^TSI^75^ motifs are critical for the convergent binding mode of IGHV1‐2 antibodies, while an “ALPPY” motif in CDR H3 results in an alternative binding mode of IGHV1‐2 antibodies [24].

IGHV1‐58/IGKV3‐20 encode a public clonotype of nAbs, first discovered by Robbiani et al. (Table 1 and Figure 4) [98]. Structural studies demonstrated that IGHV1‐58/IGKV3‐20 antibodies convergently bind to the ridge region (residues 471–491) of the SARS‐CoV‐2 RBD (Figure 3A), where the ridge accounts for approximately 75% of the entire epitope surface [57, 83, 99, 100]. Our colleagues identified an IGHV1‐58/IGKV3‐20 antibody isolated from a COVID‐19 convalescent in early 2020, CV07‐287, which binds in the same binding mode [83]. IGHV1‐58/IGKV3‐20 antibodies typically neutralize SARS‐CoV‐2 with high potency. A therapeutic antibody AZD8895 (part of the AZD8895 + AZD1061 cocktail Evusheld) belongs to this public clonotype [57]. IGHV1‐58/IGKV3‐20 antibodies neutralize a broader range of SARS‐CoV‐2 variants, including alpha, beta, gamma, and delta. However, binding and neutralization of many IGHV1‐58/IGKV3‐20 antibodies were substantially reduced against the omicron variants, including AZD8895/COV2‐2196 [26, 27, 28, 29, 31, 57, 101, 102], A23‐58.1, B1‐182.1 [100, 102], S2E12 [27, 99, 102], and COVOX‐253 [22, 103]. Importantly, IGHV1‐58/IGKV3‐20 antibodies were found to be highly elicited not only in COVID‐19 patients and vaccine recipients immunized against the ancestral SARS‐CoV‐2, but also in patients infected by the beta variant, where they adopt the canonical binding mode, highlighting the versatility of this clonotype of nAbs [83, 104]. For example, we determined the structure of an IGHV1‐58/IGKV3‐20 antibody isolated from a beta‐infected naïve convalescent, CS44, which exhibits the same binding mode as canonical IGHV1‐58/IGKV3‐20 antibodies [83]. Notably, some IGHV1‐58/IGKV3‐20 antibodies, including beta‐47 [22, 104], XGv347 [105], ZCB11 [106], P5C3 [107], and 17T2 [58] target RBD in the same mode and retain ultrapotency against the omicron variants, likely through somatic hypermutations that enhance their binding affinity and breadth. For example, somatic hypermutation enrichment analyses demonstrated that the *V*_H_ N58D mutation in CDR H2 is essential for interaction with the RBD and conferred BA.1/BA.2 neutralization activity to non‐omicron IGHV1‐58 antibodies [108].

#### 4.1.3 RBS‐C

The RBS‐C epitope is located on the right side of the RBS (Figure 3B). Many RBS‐C antibodies neutralize the ancestral SARS‐CoV‐2 with high potency. For example, we and colleagues previously reported such an antibody, CV07‐270, which was isolated from a COVID‐19 convalescent in early 2020. Our x‐ray structure demonstrated that this antibody targets RBS‐C [109]. RBS‐C antibody BD‐368‐2 (BGB‐DXP593) exhibited neutralization IC_50_ values of 1.2 and 15 ng/mL against pseudotyped and authentic SARS‐CoV‐2, respectively [78, 110]. RBS‐C antibodies are encoded by various germline genes. E484 (mutated in the beta, gamma, and omicron variants) and L452 (mutated in delta and some omicron subvariants) are located at this site and are typically critical for recognition by RBS‐C antibodies. E484 forms extensive polar interactions with RBS‐C antibodies, while L452 interacts with hydrophobic residues (e.g., CV07‐270, BD‐368‐2, P2B‐2F6, P17) [77, 78, 109, 111]. Mutations at these positions have shown the ability to evade RBS‐C antibodies [24, 32, 78, 112]. The RBS‐C site is categorized within class 2 by Barnes et al. [52].

#### 4.1.4 RBS‐D

RBS‐D is located at one end of the RBS, distant from the ridge region (Figure 3B). RBS‐D antibodies are encoded by various germline genes, and many exhibit high potency in neutralizing the ancestral SARS‐CoV‐2 (e.g., COV2‐2130/AZD1061 and REGN10987) [57, 113]. Mutations in the omicron variant at the RBS‐D site (e.g., N440K, G446S) and backbone shifts of the 371–373 loop caused by mutations S371L/F, S373P, and S375F can reduce the neutralization potency of many RBS‐D antibodies, including, but not limited to, REGN10987 and AZD1061 [26, 28, 29].

Beta‐40, Beta54, and Beta‐55 target RBS‐D and potently neutralize a broad range of SARS‐CoV‐2 variants including omicron BA.1 [22]. Y501 is present in alpha, beta, gamma, and omicron subvariants (Figure 2B). Although all three antibodies are encoded by IGHV4‐39, a germline gene overrepresented in beta‐infected patients [83, 104], Beta‐40, Beta‐54, and Beta‐55 are Y501‐specific and target RBS‐D through different angles of approach. LY‐CoV1404 (Bebtelovimab) also targets RBS‐D and straddles one corner of the RBD. LY‐CoV1404 neutralizes SARS‐CoV‐2 variants with minimal loss of potency against omicron BA.1 and BA.2 [62]. Moreover, LY‐CoV1404 showed no or little reduction in neutralizing activity against BA.2.12.1, BA.4, and BA.5 [35, 114]. However, omicron subvariants BQ.1, XBB, and later variants have evaded LY‐CoV1404 due to mutations in the epitope such as K444N/T, V445P/H, and G446S [62]. The outer RBS‐D site (closer to the N343 glycan site) is categorized within class 3 by Barnes et al. [52], while the inner RBS‐D site (closer to the CR3022 cryptic site) is within class 4.

##### 4.1.4.1 Public Antibodies Targeting RBS‐D

Bebtelovimab (LY‐CoV1404) is an FDA‐approved therapeutic mAb that potently neutralizes a broad range of SARS‐CoV‐2 variants. Another neutralizing antibody, S2X324, which retains high potency against a broad spectrum of SARS‐CoV‐2 variants, also straddles the RBD with an almost identical binding pose as LY‐CoV1404 [65]. S2X324 shares the same VH gene (IGHV2‐5) and high sequence identity with LY‐CoV1404. In fact, LY‐CoV1404 and S2X324 belong to a public antibody class encoded by IGHV2‐5/IGLV2‐14 (Table 1 and Figure 4). This class of antibodies also includes 2–7 [115], XGv265 [105], and TH027 [63]. Antibodies encoded by IGHV2‐5/IGLV2‐14 generally neutralize a broad spectrum of SARS‐CoV‐2 variants with high potency, though they are evaded by later variants.

#### 4.1.5 The CR3022 Cryptic Site

In contrast to the RBS, which is highly variable among SARS‐CoV‐2 variants and other sarbecoviruses, some other regions of the RBD are relatively conserved (Figure 3B). At the beginning of the COVID‐19 pandemic, we and others tested the binding and neutralization capabilities of SARS‐elicited mAbs against SARS‐CoV‐2 and found that CR3022 cross‐reacts with the SARS‐CoV‐2 RBD [116, 117, 118, 119]. We determined crystal structures of CR3022 in complex with the SARS‐CoV‐2 and SARS‐CoV‐1 RBDs and demonstrated that the antibody targets the same cryptic epitope (i.e., the site is not exposed in the RBD down conformation) on both viruses [116, 120]. A single mutation P384A on the CR3022 epitope fully determines the 100‐fold affinity difference against SARS‐CoV‐1 and SARS‐CoV‐2 RBDs [120]. The CR3022 site is highly conserved, with CR3022 cross‐reacting with RBDs from clade 1a, 1b, and clade 2 sarbecoviruses, including SARS‐CoV‐2 and its variants, SARS‐CoV‐1, WIV1, SHC014, RaTG13, BtKY72, pang17, Hub2013, YN2013, and Cp‐Yunnan2011 [32].

The CR3022 cryptic site does not overlap with the RBS. Therefore, most antibodies targeting this site do not directly compete with ACE2, and these antibodies generally exhibit low neutralization potency. For example, CR3022 exhibits a weak neutralization IC_50_ of approximately 10 μg/mL against SARS‐CoV‐1 [121] and shows little to no neutralization activity against SARS‐CoV‐2 [116, 117]. An affinity‐matured antibody derived from CR3022 using yeast display, named eCR3022.20, demonstrated improved binding and neutralization against SARS‐CoV‐2 [122]. Our structural study showed that eCR3022.20 binds to the same epitope and with the same angle of approach as CR3022, with mutated paratopic residues facilitating enhanced binding and neutralization against SARS‐CoV‐2 [122]. Antibodies such as S304 and COVA309‐22 also target a similar region with limited direct competition with ACE2 binding. While these antibodies exhibit broad and strong binding to SARS‐CoV‐2, SARS‐CoV‐1, and other sarbecoviruses, they have low neutralization activity [44, 123]. The CR3022 cryptic site is categorized as class 4 by Barnes et al. [52].

##### 4.1.5.1 A D‐Gene‐Determined Public Antibody Class With YYDxxG Motif in CDRH3

**FIGURE 5:**
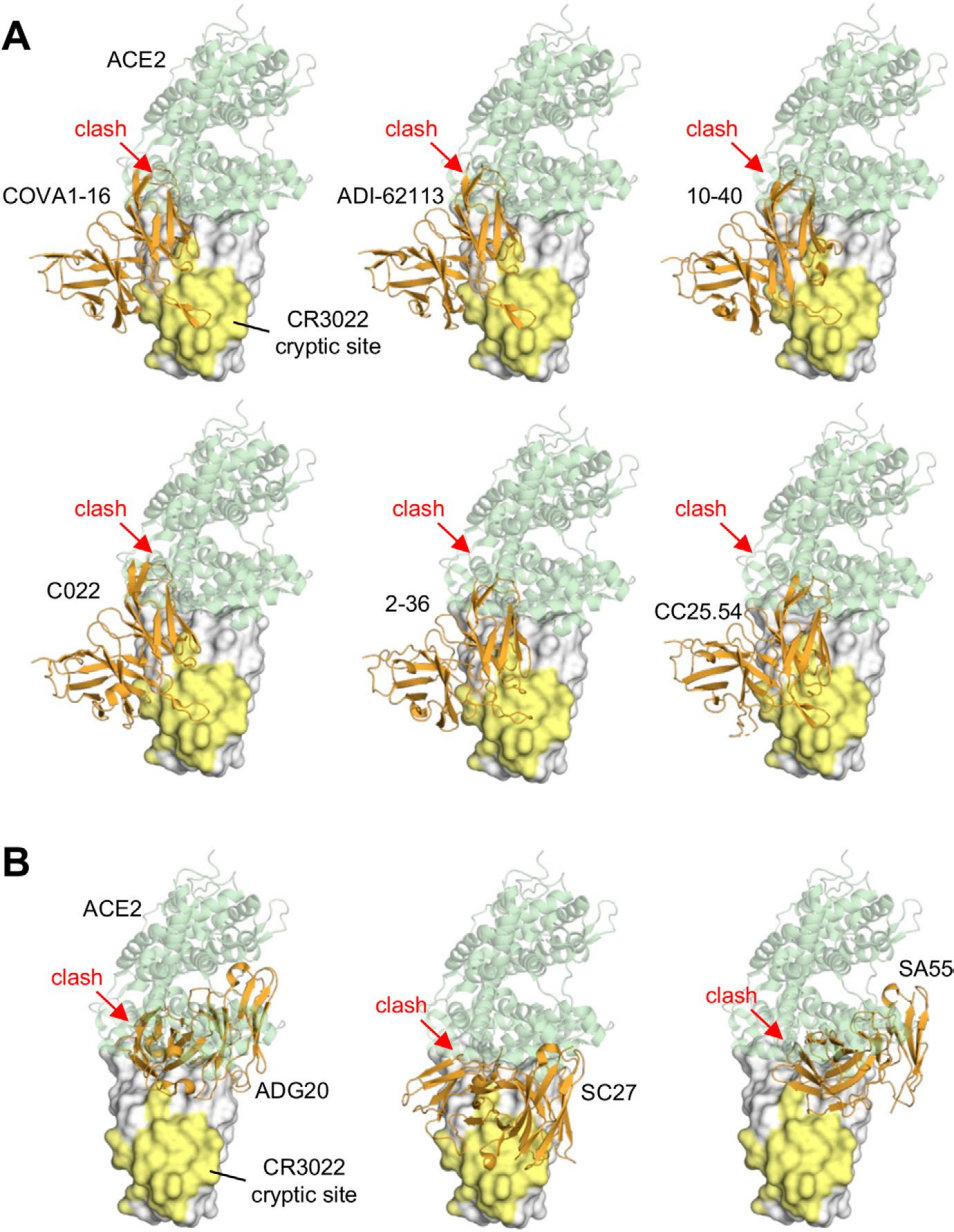
Antibodies targeting the conserved CR3022 cryptic site that clash with ACE2 binding. (A) Structurally convergent public antibodies with the “YYDxxG” motif in the CDR H3 target the conserved CR3022 cryptic site, while their angles of approach enable blocking of binding to angiotensin‐converting enzyme 2 (ACE2). (B) Antibodies targeting the RBS‐D/CR3022 (Class 1/4) site. The epitopes span from a corner of the RBS to the conserved CR3022 cryptic site. The SARS‐CoV‐2 RBD is shown as a white surface with the CR3022 site highlighted in yellow, while the variable domains of the antibodies are in orange. The RBD–antibody structure is superimposed onto an RBD‐ACE2 structure (PDB 6M0J) and illustrates that the antibodies would clash (red arrows) with ACE2 (green). The PDB codes of the antibody/antigen complex structures shown here are: COVA1‐16: 7JMW; ADI‐62113: 7T7B; 10–40: 7SD5; C022: 7RKU; 2–36: 7N5H; CC25.54: 8SIR; ADG20: 7U2D; SC27: 8VIF; SA55: 7Y0W.

Due to the nonoverlapping nature of the CR3022 site and the RBS, some antibodies targeting the CR3022 site do not directly compete with ACE2 binding and generally neutralize with low potency, despite high binding affinity. However, we and others have identified a class of antibodies containing a YYDxxG motif in ultralong CDR H3 loops (19–26 residues; e.g., COVA1‐16, ADI‐62113, 10–40, C022, etc.) that target this highly conserved non‐RBS site with a unique angle of approach, creating steric hindrance with ACE2 binding, although the antibody and ACE2 epitopes do not overlap (Table 1, Figures 4 and 5). These antibodies elicit potent neutralization against SARS‐CoV‐2 variants and other sarbecoviruses [124, 125]. Additionally, this special angle of approach facilitates bivalent binding and, therefore, increases avidity, enhancing the potency of the IgG [66]. In contrast, antibodies that bind the same site with a more perpendicular angle, such as CR3022, exhibit a much weaker avidity effect and, thus, more limited neutralization titers [66, 120, 125].

YYDxxG‐containing antibodies have retained neutralization activity against omicron variants, although with some reduction [29, 31, 68]. The structural convergence of most public antibodies is determined by their shared immunoglobulin V genes. We demonstrated that this class of antibodies uses the YYDxxG motif in CDR H3 to form extensive interactions with the antigen, all exclusively encoded by IGHD3‐22, while the V and J genes vary, confirming a D‐gene‐specific public class of antibodies [67]. This example is similar to our previous observation of D3‐9‐encoded regions dominating antibody interactions with the influenza A HA stem [126]. The observation of the D‐gene‐dominating public antibody classes demonstrates a great potential for D‐gene‐based, germline‐targeting vaccine design.

##### 4.1.5.2 A Light Chain‐Defined Public Antibody Class

While the structural convergence of most public antibodies is typically determined by heavy chain germlines, we have reported a public antibody response to the SARS‐CoV‐2 S protein defined by shared light chain usage, specifically encoded by IGLV6‐57, along with conserved CDR H3 regions that predominantly interact with the SARS‐CoV‐2 RBD, with varying IGHV genes [11]. For example, the light chain of S2A4, an IGLV6‐57 nAb, contributes 53% of the BSA of the epitope, while CDR H3 contributes 38% [44]. The hydrophobic CDR H3, usually containing a ^97^WL/VRG^100^ motif, forms extensive interactions with the RBD. The public class of antibodies target the CR3022 cryptic site (Figure 4). IGLV6‐57 nAbs have been further explored in studies such as Yan et al. [127], where six mAbs were isolated from COVID‐19 convalescents by phage display in early 2020. Notably, five out of the six mAbs were encoded by IGLV6‐57. Among these, R1‐30, R2‐3, R2‐6, and R2‐7 shared the “WLRG” motif in CDR H3, while R1‐26 showed a “LGPWV” motif, targeting the RBD in a same binding mode (Figure 4). The antibodies were also escaped by omicron‐specific mutations at residues S371, S373, and S375 [127, 128]. Other IGLV6‐57 antibodies with hydrophobic CDR H3s, such as P5S‐3B11 [129], 3D11 [130], H18 [127], WRAIR‐2151 [131], IY‐2A [132], FP‐12A [132], 002‐13 [133], 553‐15 [134], and 2‐7 [135], have also been reported to target the RBD in the shared mode. The identification of the IGLV6‐57‐encoded antibody class demonstrates that light chains can play an essential role in the public antibody response.

#### 4.1.6 The RBS‐D/CR3022 Site: A Region That Spans Two Sites

Some antibodies target a site on the RBD that spans both the RBS‐D and CR3022 sites, known as the RBS‐D/CR3022 site. Antibodies such as ADG20 [32, 136] (Figures 3B and 5) and its affinity‐matured derivative VYD222 target this region [137]. These antibodies take advantage of the properties of both sites: targeting the RBS region enables direct competition with ACE2 binding, providing strong neutralization potency, while targeting the conserved region offers broad protection not only against SARS‐CoV‐2 variants, including omicron, but also against other sarbecoviruses [32]. The RBS‐D/CR3022 site is also referred to as class 1/4 [138]. Other groups have identified antibodies targeting this site, including SA55 [139, 140], SC27 [141], and BD55‐4637 [139, 142], which exhibit exceptionally strong neutralization potency and breadth. SC27 has been reported to neutralize recent SARS‐CoV‐2 variants JN.1 and HV.1. SA55 is able to neutralize JN.1.1, KP.1.1, LB.1, and KP.3.3 with strong potency. Overall, the RBS‐D/CR3022 site holds great promise for developing broad and potent antibodies that are resistant to immune escape. More details about antibodies targeting this site will be discussed next in the section ‘Developing escape‐resistant potent neutralizing antibodies against SARS‐CoV‐2’.

#### 4.1.7 The N343 Glycan Site

The N343 glycan is conserved across all SARS‐CoV‐2 variants and the *Sarbecovirus* subgenus [143]. This site is categorized as class 3 by Barnes et al. [52] Sztain et al. revealed that the N343 glycan plays a critical role in viral entry, where the glycan gate facilitates RBD opening. A weighted ensemble (WE) simulation study demonstrated that the glycan initiates the RBD “down‐to‐up” transition by intercalating between residues of the ACE2 binding motif [144]. The N343 glycan is recognized and forms important interactions with many antibodies, such as S309 [143], CV38‐142 [145], and CC6.33 [146]. S309 is a mAb isolated from a SARS‐CoV‐1 patient that cross‐reacts with SARS‐CoV‐2 by targeting the N343 glycan site [143]. This site is located on the opposite lateral face of the RBD from the CR3022 cryptic site (Figure 3B). CV38‐142 [145] and CC6.33 [146, 147] were isolated from SARS‐CoV‐2‐infected patients, which have also been reported to bind to the N343 glycan site and cross‐neutralize both SARS‐CoV‐2 variants and SARS‐CoV‐1. Although CC6.33 neutralizes ancestral SARS‐CoV‐2 with high potency, it failed to neutralize the omicron BA.1 variant [146]. S309 and its derivative sotrovimab have been reported to neutralize ancestral SARS‐CoV‐2 with medium potency and retain neutralizing activity against the omicron BA.1 variant, with only a 2.5‐fold reduction in potency [29]. This neutralization potency was reduced by 27‐fold against BA.2 [31]. mAbs BD55‐5840 (SA58) and BD55‐3546 were isolated from SARS‐CoV‐1 convalescent patients, also targeting the N343 glycan site [148]. SA58 broadly neutralizes SARS‐CoV‐2 variants, including omicron subvariants BA.4/5 and BQ.1, but was evaded by XBB and later variants. SA58 and BD55‐3546 are both encoded by IGHV7‐4‐1/IGKV3‐15, suggesting a rare but important antibody class with remarkable neutralization potency and breadth.

#### 4.1.8 RBD Site V

Compared to antibodies targeting other regions of the RBD (RBS, CR3022 cryptic site, and N343 glycan site), which were discovered at the beginning of the COVID‐19 pandemic, site V antibodies were identified later. Site V antibodies were first reported in 2021, including S2H97 [149] and COVOX‐45 [150]. Additionally, monoclonal antibodies N‐612‐056 [151], ION_300 [152], 6D6 [153], XMA09 [154], WRAIR‐2063 [155], WRAIR‐2134 [156], and IMCAS74 [157] also target site V. We also reported structures of three highly broad nAbs targeting this site, CC25.4, CC25.43, and CC25.56 [124, 158]. These antibodies were isolated from vaccinated COVID‐19 convalescents using SARS‐CoV‐1 and SARS‐CoV‐2 spike proteins as baits. Site V is a non‐RBS cryptic site located beneath the ridge region (Figure 3). The binding of site V antibodies requires extensive opening of the RBD to expose the epitope and prevent clash of the antibodies with the adjacent protomers of S. For example, the binding of S2H97 causes sufficient steric clashes and S trimer destabilization that the SARS‐CoV‐2 S protein is induced to refold into the postfusion state, promoting S1 shedding [149]. Site V antibodies exhibit relatively modest neutralization potency compared to RBS‐targeting antibodies, but the site is highly conserved, and nAbs targeting this region usually exhibit exceptionally broad neutralization effects. For example, S2H97 demonstrated a broad range of binding to coronavirus RBDs (from clade 1a to clade 3) and was insensitive to SARS‐CoV‐2 variants, including omicron [27]. ION_300 also neutralizes recently emerged omicron subvariants BA.2.86 and JN.1 [91].

#### 4.1.9 NTD Antigenic Supersite

The NTD (residues 14–306) is located at the N‐terminal region of the SARS‐CoV‐2 S protein (Figure 1). The NTD is more variable compared to the RBD (other than the RBS; Figure 2B). The amino acid sequence identity between ancestral SARS‐CoV‐2 and SARS‐CoV‐1 NTDs is 53%, lower than that of the RBD (73%) [159]. Anti‐NTD neutralizing antibodies primarily target the NTD antigenic supersite (also referred to as site i [46]), which consists of variable loops flanked by glycans at N17 and N149 [46, 69, 160, 161, 162, 163, 164, 165, 166]. This site, located on one side of the NTD, is also distal to the viral membrane as is the RBD. Antibodies targeting this site can exhibit high neutralization activity, comparable to some RBS antibodies [160].

##### 4.1.9.1 Public Antibodies Targeting the NTD Antigenic Supersite

IGHV1‐24 antibodies are highly over‐represented in targeting this site (Table 1, Figures 3C and 4). For example, nAbs such as 4A8 [69], CM25 [165], DH1050.1 [162], 1–87, 2–51 [160, 161], and TXG‐0078 [70] are encoded by IGHV1‐24 and target the antigenic supersite with a convergent binding mode. Additionally, antibodies encoded by various germline genes have also been reported to target the antigenic supersite and can exhibit excellent neutralization potency, including S2L28 [46], 2–17, 4–18 [160, 161], S2M28, S2X28, S2X333 [46], DH1051 [162], and CoVIC‐246 [165].

Neutralizing antibodies targeting the NTD supersite are largely evaded by SARS‐CoV‐2 mutations as well as deletions. Most tested nAbs targeting this site showed substantially reduced binding to alpha, beta, and delta variants [46, 167]. The Y144 deletion (present in alpha and in omicron subvariants later than XBB) caused a substantial reduction in supersite antibodies [46]. The S13I and W152C mutations in the epsilon variant induced a shift in the signal peptide cleavage site and the formation of a new disulfide bond, resulting in the total loss of neutralization for antibodies targeting the NTD antigenic supersite [168]. Omicron BA.1 also escaped from antibodies targeting the NTD supersite due to del143–145 and G142D, which are incompatible with the binding of several potent supersite nAbs [29, 169]. TXG‐0078, an IGHV1–24‐encoded mAb, binds the NTD supersite region of the S protein and recognizes a diverse collection of alpha‐ and beta‐coronaviruses [70]. TXG‐0078 achieves its binding breadth through an ultralong CDR H3 compared to other NTD supersite‐specific nAbs by making additional contacts with the NTD compared to other IGHV1‐24 antibodies. In addition, TXG‐0078 has a unique V_H_ F96 located at an untemplated position (N and P additions) on its CDR H3 and forms an interaction with P251 on the NTD that is conserved across all TXG‐0078‐reactive betacoronaviruses, including SARS‐CoV‐2 WA1, beta, gamma, kappa, HCoV‐OC43, and HCoV‐HKU1 [70]. In summary, the NTD antigenic supersite is targeted by many nAbs with high neutralization potency, but its high variability among variants largely results in immune escape from these nAbs.

#### 4.1.10 NTD Site iv

Site iv is a relatively conserved site on the NTD, located on the opposite face from the supersite (Figure 3C). It is on the inner side of the NTD, close to RBD within the same protomer. Antibody S2L20 binds to site iv [46] and interacts with a wide range of SARS‐CoV‐2 variants, including alpha, beta, gamma, delta [167], and omicron BA.1 [169]. However, S2L20 exhibited little neutralization activity against SARS‐CoV‐2 [46]. Another antibody, C1791, binds to a similar site as S2L20 and showed weak neutralization against gamma and omicron BA.1 variants [170].

#### 4.1.11 A Hydrophobic Site on the Back of the NTD

Several mAbs have been found to bind a hydrophobic site on the back of the NTD, that is, the opposite side of the supersite (Figure 3C). mAbs such as S2X303 [46], 5–7 [171], P008‐056 [172, 173], and C1520 [170] target this site with high neutralization potency and broader neutralization spectrum compared to the supersite‐targeting nAbs. While supersite antibodies are sensitive to mutations in most variants, some mAbs targeting this hydrophobic site retain activity against certain variants. For example, S2X303 retains activity against beta, gamma, and delta variants, but is sensitive to alpha and omicron BA.1 [27, 167], presumably due to its interaction with Y144 that was deleted in these variants. Similarly, 5–7 tolerates mutations in alpha, beta, and omicron BA.1 but is sensitive to gamma, delta, and omicron BA.2 [29, 171, 174]. C1520 retains strong activity against alpha, beta, delta, and omicron BA.1, and was evaded by XBB [170, 175].

#### 4.1.12 NTD Sites v and vi

NTD site v is located at the peripheral‐most part of the NTD (Figure 3C). Antibody S2X316 targets NTD site v, interacting with an epitope near the glycans at N74 and N149, but exhibits no neutralization activity despite its medium binding affinity [46]. NTD site vi is located on the side of the NTD that is more proximal to the viral membrane (Figure 3C). Antibodies such as DH1052 were reported to target this site with high binding affinity, but similarly showed no neutralization activity [46, 162].

#### 4.1.13 SD1 and SD2

The SD1 domain is relatively more conserved compared to the NTD and RBD (Figure 3A). Mutations at three positions have been observed in SARS‐CoV‐2 major variants. T547K emerged in BA.1 but not BA.2 and later variants. E554K and A570V emerged in BA.2.86 and later variants. A570D was also found in the alpha variant. S3H3, an antibody isolated from S‐immunized mice, was shown to target SD1 and broadly neutralize SARS‐CoV‐2 and variants including alpha, beta, delta, omicron subvariants BA.1, BA.2, as well as later variants XBB.1, EG.5.1, and HK.3, with high neutralization potency [176, 177, 178, 179]. Another antibody, SD1‐1, was also found to target SD1 and neutralize a broad range of SARS‐CoV‐2 variants [180]. The neutralization of S3H3 and SD1‐1 was largely reduced against BA.2.86 and JN.1 [178, 180], where the single mutation E554K in these variants led to a complete knock out of S3H3 and SD1‐1 [180, 181]. Human antibody C1717 targets a neutralizing site on the membrane‐proximal side of the NTD, bridging the SD2 domain on the same protomer, and neutralizes SARS‐CoV‐2 variants including alpha, beta, gamma, and omicron subvariants, albeit with medium neutralization potency [142, 170].

### 4.2 S2 Domain

#### 4.2.1 Fusion Peptide

**FIGURE 6:**
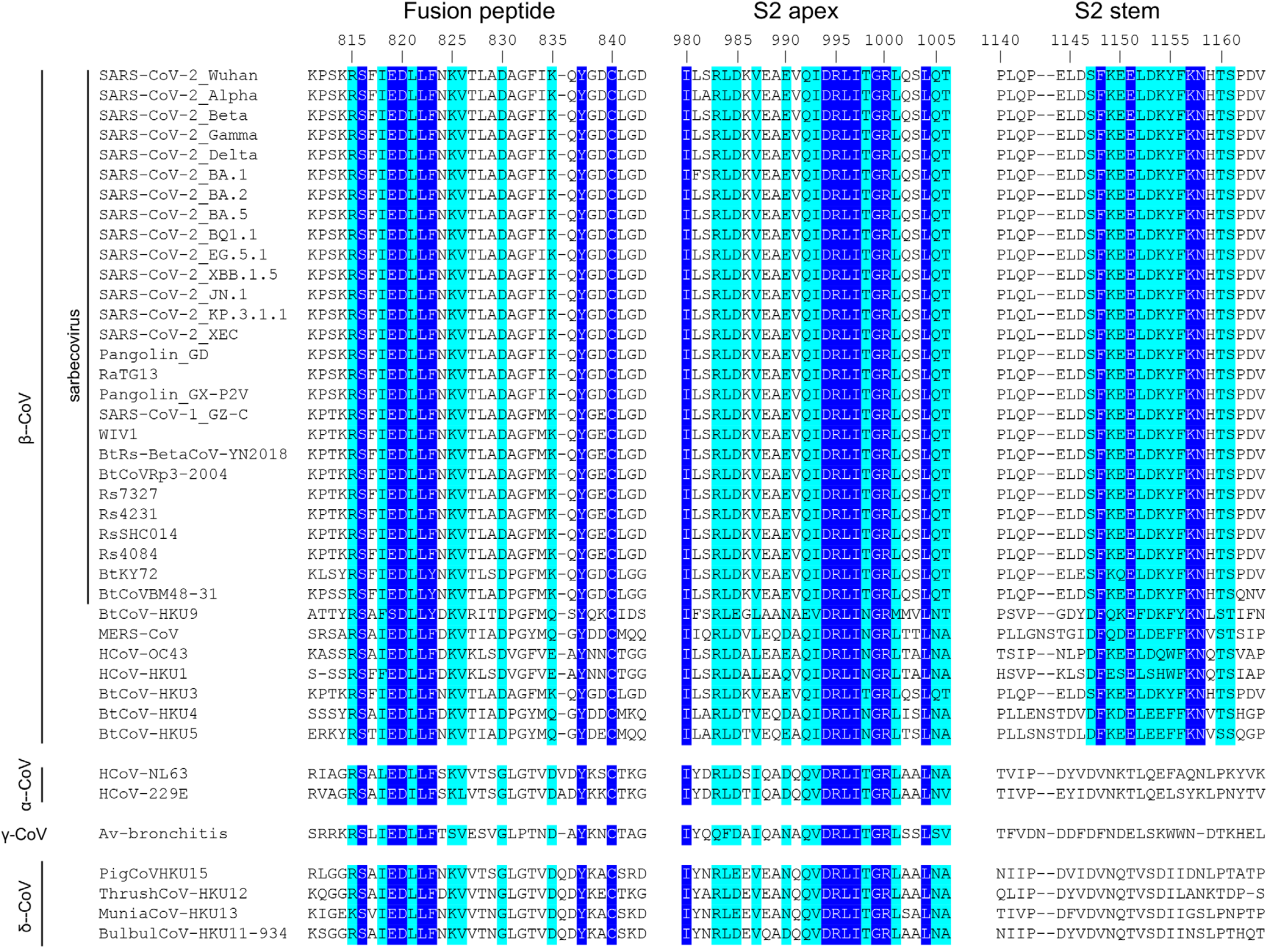
Sequence alignment of the fusion peptide, S2 apex, and S2 stem regions across coronaviruses. Amino acids in blue boxes are positions which have a single, fully conserved residue, while cyan boxes indicate conservation between groups of similar properties (scoring > 0 in the Gonnet PAM 250 matrix) [183]. The sequence alignment was performed using Clustal Omega [3]. The fusion peptide and S2 apex are conserved across all genera of coronaviruses, while the S2 stem region is conserved across betacoronaviruses where sequence conservation colors are only shown for the betacoronaviruses.

SARS‐CoV‐2 fusion peptide (FP) is located immediately after the S2/S2’ cleavage site (Figure 1). Upon engagement of the RBD with the host cell receptor ACE2 and subsequent proteolytic cleavage by TMPRSS2 at the cell surface or cathepsins in endolysosomes, the S1 subunits dissociate from the S protein, which then undergoes a large structural rearrangement that exposes the fusion peptide. The fusion peptide inserts into host cell membranes, facilitating viral fusion [182]. The fusion peptide is highly conserved among the Orthocoronavirinae subfamily, including alphacoronaviruses, betacoronaviruses, gammacoronaviruses, and deltacoronaviruses (Figure 6). No mutations have been identified in the fusion peptide region of SARS‐CoV‐2 variants of concern. Other antibodies targeting the fusion peptide have been reported, showing low‐to‐medium neutralization activity against SARS‐CoV‐2 and variants, isolated from COVID‐19 convalescents and vaccinees, including 76E1 [184], C77G12, VN01H1, VP12E7 [9], COV44‐62, COV44‐79, COV91‐27 [8], fp.006 [185], and C20.119 [186]. Remarkably, 76E1 [184], C77G12, VN01H1, VP12E7 [9], COV44‐62, COV44‐79, and COV91‐27 [8] have been reported to demonstrate broad neutralization activity against non‐sarbecoviruses, such as MERS‐CoV (betacoronavirus), OC43 (betacoronavirus), NL63 (alphacoronavirus), and 229E (alphacoronavirus). A non‐neutralizing antibody DH1058 has been also found to broadly react with the fusion peptides of SARS‐CoV‐2, SARS‐CoV‐1, MERS‐CoV, 229E, NL63, HKU1, and OC43 [162, 187]. Structural studies showed that the epitopes on the fusion peptide recognized by these nAbs are usually cryptic in the prefusion state of the S protein, suggesting that a conformational change or conformational dynamics around the fusion peptide is required to accommodate the nAbs. Lei et al. performed deep mutational scanning (DMS) experiments on an S2 region spanning the fusion peptide and demonstrated that the F823Y mutation, present in the bat betacoronavirus HKU9 spike protein, confers resistance to broadly neutralizing antibodies, suggesting a potential future mutation of SARS‐CoV‐2 to potentially escape anti‐FP antibodies [188].

#### 4.2.2 S2 Apex

The S2 apex region (residues 980–1006) is located at the upper part of the S2 subunit of the prefusion S protein (Figure 3A). This epitope is highly conserved across different genera of coronaviruses (Figure 6). The S2 apex is typically hidden in the closed conformation of the spike protein and becomes exposed when the S protein transitions to an open conformation [189, 190]. Monoclonal antibodies targeting the S2 apex region have been isolated from mice (3A3 and RAY53 [191]) and a convalescent COVID‐19 donor (54043‐5 [192]). These antibodies exhibit highly broad reactivity. Despite limited neutralization activity, S2 apex‐targeting antibodies mediate Fc effector functions. In particular, an in vivo study showed that the Fc effector functions of 54043‐5 confer neutralization independent protective effects in mice [192].

##### 4.2.2.1 Public Antibodies Targeting the S2 Apex

Despite usually being non‐neutralizing, antibodies targeting the S2 apex are dominant in the B‐cell population following SARS‐CoV‐2 infection. A public class of B cells targeting the S2 apex, encoded by IGHV1‐69/IGKV3‐11, was found to recognize the S2 apex region of SARS‐CoV‐2 (Figure 4) [72, 186]. Additionally, IGHV1‐69/IGKV3‐11 B cells are highly enriched in the naïve human repertoire and confer protection in vivo through Fc‐mediated effector functions. A recent study found that D950N and Q954H, observed in delta and omicron variants, weakened the binding of IGHV1‐69/IGKV3‐11 antibodies to the S protein [71].

#### 4.2.3 S2 Stem Helix

The S2 stem helix is located at the C terminus of the SARS‐CoV‐2 S2 subunit ectodomain (residues 1141–1162; Figure 1A). In the prefusion state of SARS‐CoV‐2 S trimer, the S2 regions form a 3‐helix bundle at the bottom of the S protein (Figures 1A and 3A). The S2 stem region is highly conserved among betacoronaviruses, including sarbeco‐ and non‐sarbecoviruses, such as MERS‐CoV and HKU1 (Figures 3A and 6). While mutations have been found in the RBD, NTD, and other parts of the S2 subunit, no mutations have been detected in the S2 stem region in SARS‐CoV‐2 variants as of October 2024.

We previously characterized structures of antibodies targeting the SARS‐CoV‐2 S2 stem helix isolated from COVID‐19 convalescents, including CC40.8 [193, 194], CC25.106, CC95.108, CC68.109, CC99.103 [7], COV30‐14, COV89‐22, and COV93‐03 [73]. Other nAbs have also been reported to target the S2 stem and neutralize SARS‐CoV‐2, including S2P6 [10] and CV3‐25 [195, 196, 197], elicited in human COVID‐19 patients, as well as B6, IgG22, and WS6, isolated from spike‐immunized mice [198, 199, 200]. Antibodies targeting the S2 stem helix generally cross‐react with and broadly neutralize a wide range of betacoronaviruses, including SARS‐CoV‐2 and variants, SARS‐CoV‐1, HCoV‐HKU1, and MERS‐CoV. The neutralization activity of anti‐S2 stem antibodies is typically modest.

##### 4.2.3.1 Public Antibodies Targeting the S2 Stem helix

Many nAbs from different donors targeting the S2 stem region are encoded by human IGHV1‐46/IGKV3‐20 (e.g., S2P6 [10], CC68.109, CC99.103 [7], COV30‐14, COV89‐22, and COV93‐03 [73]). They all bind to the S2 stem helix using the same binding mode (Figure 4), demonstrating that these antibodies belong to a frequent public class of antibodies. A PPxF motif is commonly found in CDR L3 of IGHV1‐46/IGKV3‐20 anti‐S2 antibodies, and acquired through VJ recombination. Our structural study demonstrated that the PPxF motif forms essential interactions for the recognition of the S2 stem helix [7]. We also identified another public class of S2 stem‐targeting antibodies encoded by IGHV1‐46/IGLV1‐51 (Figure 4) [7].

Intriguingly, almost all antibodies targeting the S2 stem helix bind to the same conserved hydrophobic interface located at the protomer–protomer interface of a prefusion S molecule (Figure 3A). This observation suggests that binding of an S2‐stem‐targeting antibody would clash with an adjacent protomer of prefusion S. Recently, a cryoelectron tomography (cryoET) study captured an intermediate state of S2 refolding, suggesting that antibodies targeting the S2 stem helix involve binding to the prehairpin intermediate of the SARS‐CoV‐2 S protein [201]. These antibodies prevent the spike from undergoing its conformational transition necessary for membrane fusion by sterically blocking the “back‐zippering” of the HR2 domain onto HR1. This inhibition stabilizes the prehairpin intermediate, halting the fusion process, and preventing the viral and host membranes from coming closer together, thereby neutralizing the virus's infectivity.

## 5 Summary of Neutralizing Epitopes on SARS‐CoV‐2

**FIGURE 7:**
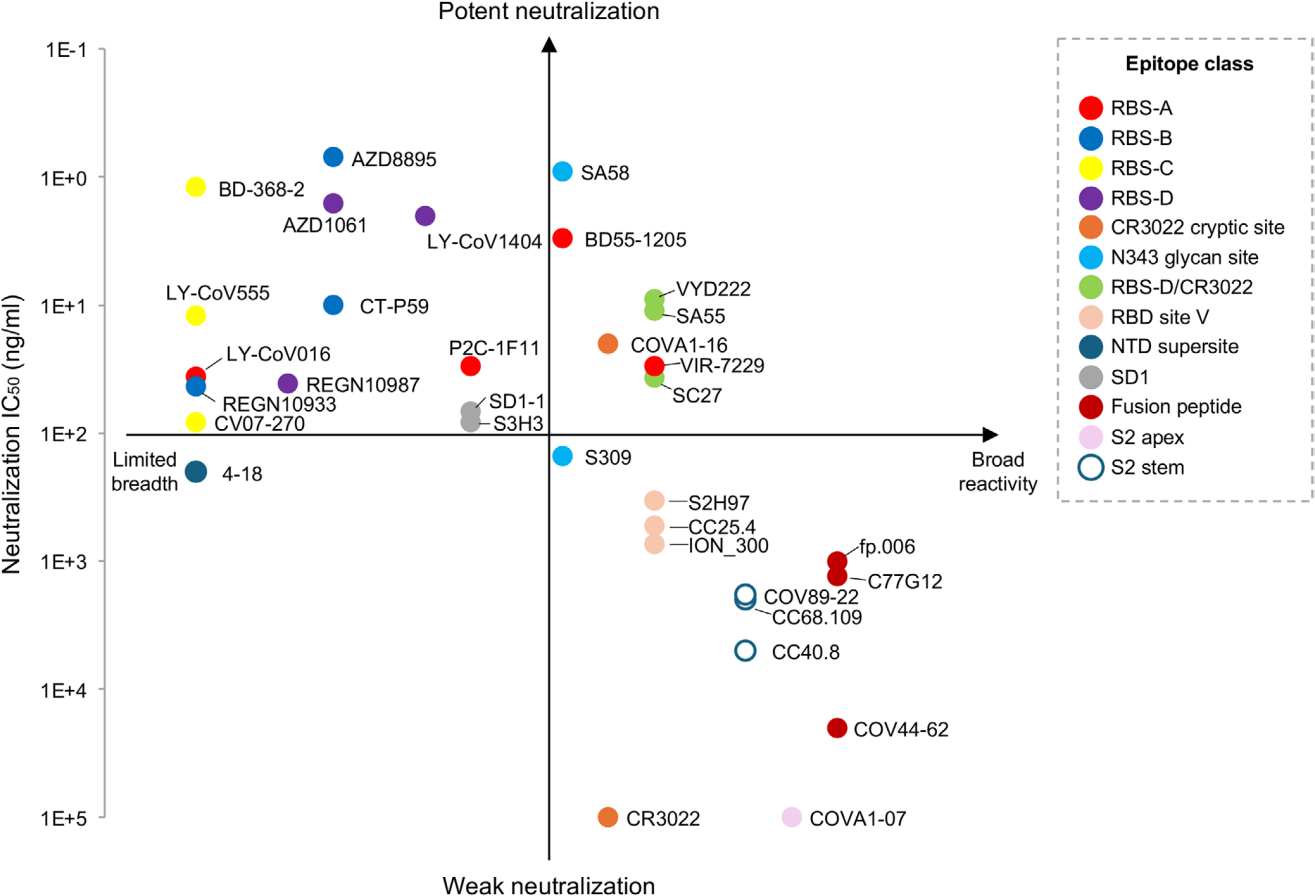
An overview of the breadth and potency of mAbs targeting the SARS‐CoV‐2 S protein. The neutralizing IC_50_ values are to the wild‐type SARS‐CoV‐2 from the original papers of each mAbs and shown along the *y*‐axis. The reactive breadth of binding on the *x*‐axis includes cross‐reactivity to SARS‐CoV‐2 variants, sarbecoviruses, betacoronaviruses, and other genera of coronaviruses. Note that neutralizing IC_50_ values are from different groups and are only comparable in terms of order of magnitude.

We have reviewed mAbs targeting various epitopes throughout the SARS‐CoV‐2 S protein. Many neutralizing mAbs are highly potent against the ancestral SARS‐CoV‐2 but lose potency against variants due to mutations at variable epitopes, such as the RBS. In contrast, antibodies targeting relatively conserved sites, like the fusion peptide, are more resistant to escape and are generally less affected by SARS‐CoV‐2 evolution, although they neutralize with relatively lower potency. In these cases, neutralization potency and breadth are often mutually exclusive (Figure 7). However, during the analysis of SARS‐CoV‐2 antibodies, we noticed some rare but recurring antibodies targeting certain sites on SARS‐CoV‐2 S protein that exhibit minimal loss of activity against variants. These antibodies are discussed in the next section.

## 6 Developing Escape‐Resistant Potent Neutralizing Antibodies Against SARS‐CoV‐2

Previous efforts have identified several B‐cell epitopes and numerous monoclonal antibodies against SARS‐CoV‐2. However, the human immune response and many neutralizing antibodies have been largely evaded by the extensive mutations that occurred during SARS‐CoV‐2 evolution. Nearly all FDA‐approved therapeutic antibodies have been evaded by SARS‐CoV‐2 variants. Variants of SARS‐CoV‐2 continue to circulate globally, posing ongoing risks to public health and driving further viral evolution. Developing escape‐resistant potent neutralizing antibodies is essential for the treatment of infections caused by current and future SARS‐CoV‐2 variants. Additionally, creating such antibodies may offer a blueprint for developing strategies against other highly variable viruses.

One of the main challenges in developing escape‐resistant potent neutralizing antibodies against SARS‐CoV‐2 is that potency and breadth are often mutually exclusive. In many viruses, receptor‐binding sites (RBSs) are relatively, or at least moderately, conserved regions—such as the hemagglutinin (HA) in influenza [202, 203] and the envelope protein (Env) in HIV [204]—since these regions play a crucial role in host receptor binding, and substantial mutations may impair this vital function. In contrast, the RBS of SARS‐CoV‐2 presents a notable exception—it remains one of the most variable regions of the S protein, capable of undergoing substantial evolutionary changes with minimal impact so far on viral fitness, all while effectively evading nAbs generated by natural infection or vaccination. As discussed earlier, antibodies targeting the RBS sites (i.e., RBS‐A, RBS‐B, RBS‐C, and RBS‐D) generally demonstrate highly potent neutralizing activity, likely due to their direct receptor‐blocking effect. The rapid mutation of RBD residues during SARS‐CoV‐2 evolution enables the virus to evade most RBS‐targeting antibodies and limit their neutralization breadth. Similarly, the NTD is highly variable, and antibodies targeting the supersite typically exhibit limited cross‐reactivity with SARS‐CoV‐2 variants. These regions accommodate antibodies that are generally potent but not broad. In contrast, several non‐RBS sites are relatively more conserved and targeted by neutralizing antibodies. For example, the CR3022 cryptic site and RBD site V are conserved across sarbecoviruses, while the S2 stem helix is conserved across betacoronaviruses, and the fusion peptide is conserved across different genera of coronaviruses (Figure 6). These minimally changed regions suggest high functional conservation and are rarely mutated during SARS‐CoV‐2 evolution. Antibodies targeting these sites are broadly reactive but tend to exhibit only low‐to‐moderate neutralizing potency (i.e., broad but not potent).

A main goal in developing SARS‐CoV‐2 therapeutics is to create escape‐resistant potent neutralizing antibodies (i.e., broad‐and‐potent nAbs). A structurally convergent public class of antibodies, characterized by a common IGHD3‐22‐encoded “YYDxxG” motif in the CDR H3, target the conserved CR3022 cryptic site, which does not overlap with the RBS. These antibodies bind the S protein at a unique angle, allowing for direct inhibition of ACE2 binding (Figure 5A), thereby conferring both strong neutralization potency and broad reactivity. However, despite the limited mutations observed at the epitopes of YYDxxG antibodies, their neutralization activity has been reduced against recent SARS‐CoV‐2 Omicron subvariants [205, 206]. This reduction is likely due to the more tightly packed prefusion S trimer, with RBDs more stabilized in down conformations [207], effectively hiding the epitope.

We previously identified a site that spans the right corner of the RBS and the CR3022 region (Figures 2 and 5B), referred to as the RBS‐D/CR3022 site [32]. This site is also known as class 1/4, as it overlaps with class‐1 and class‐4 epitopes [35]. Antibodies targeting this site capitalize on both potency, derived from the RBS, and breadth, provided by the conserved CR3022 site. Remarkably, a few antibodies, such as VYD222 [208], SA55 [35, 139, 140], and SC27 [141], exhibit exceptionally strong neutralization potency and breadth. These antibodies cross‐react with a wide range of sarbecoviruses and neutralize SARS‐CoV‐2 variants with high efficacy. Notably, VYD222 and SA55 have been shown to neutralize the latest SARS‐CoV‐2 variants, KP.3 and KP.3.1.1, as of October 2024. Overall, the RBS‐D/CR3022 site represents a highly promising target for the development of broad and potent antibodies resistant to immune escape.

VYD222 has received FDA EUA for pre‐exposure prophylaxis (PrEP) of COVID‐19. Notably, the pan‐sarbecovirus neutralizing antibody VYD222 is derived from a human anti‐SARS‐CoV‐1 mAb, ADI‐55688, with intermediate mAbs including ADG20 [32] generated through in vitro affinity maturation [136]. ADI‐55688, isolated from a SARS convalescent exhibited modest cross‐neutralization activity against SARS‐CoV‐2 [209]. Each step of maturation improved either binding affinity or broadened neutralization. For example, the transition from ADI‐55688 to ADG20 involved five amino acid mutations, resulting in nearly a 200‐fold increase in binding affinity and a 100‐fold improvement in neutralization potency against SARS‐CoV‐2 [136]. ADG20 effectively neutralized omicron BA.1, but not BA.2 and subsequent variants. Remarkably, further affinity maturation from ADG20 to VYD222 largely expanded neutralization breadth, enabling VYD222 to neutralize not only omicron BA.1 but all circulating variants, including the latest KP.3.1.1 [137]. Other exceptionally broad and potent nAbs include AZD3152, matured from Omi‐42 [92, 93], and VIR‐7229, derived from S2V29 [210]. The extensive enhancements observed through affinity maturation highlight the potential of this process to optimize antibodies targeting key sites, providing both potency and breadth for developing escape‐resistant neutralizing antibodies against SARS‐CoV‐2.

Despite being highly variable, RBS‐A is targeted by an exceptional antibody BD55‐1205, which belongs to a public class of antibodies encoded by IGHV3‐53/3‐66. This germline is one of the most canonical and frequently elicited antibody classes, typically targeting variable epitopes within the RBS‐A site. As a result, IGHV3‐53/3‐66 antibodies are often evaded by SARS‐CoV‐2 variants due to mutations. However, BD55‐1205 is a rare IGHV3‐53/3‐66 antibody that demonstrates potent neutralization against all known SARS‐CoV‐2 variants, including the currently predominant variants KP.3 and KP.3.1.1. A germline‐revertant study showed that somatic hypermutations and its particular CDR H3 may jointly contribute to the extraordinary breadth of BD55‐1205 [55]. This exception underscores the promising potential of this antibody germline. Importantly, the global population is now largely imprinted by prior infection or vaccination that is primed by prototype SARS‐CoV‐2, which would generate IGHV3‐53/3‐66 B cells with high frequency, while some B cells within this class, though rare, retain activity in breakthrough infections by BA.1, BA.5, HK.3, or JN.1 [81, 88, 211, 212]. Finding a trajectory that directs the primed population to a matured IGHV3‐53/3‐66 B‐cell like BD55‐1205 will be invaluable for public immunity against current and future SARS‐CoV‐2 variants.

The global population's immunity has been predominantly shaped by exposure to the ancestral strain of SARS‐CoV‐2. As a result, the immune memory of the world is primarily imprinted by these early strains. However, de novo activation of omicron‐specific B cells, though rare, has been observed in individuals exposed to antigens that are antigenically distinct from the original Wuhan strain, such as BA.1 or JN.1 [212, 213, 214, 215]. In addition, priming with omicron subvariants XBB and JN.1 in naïve humans elicited distinct nAbs with minimal cross‐reactivity with Wuhan suggesting a large antigenic gap, and resulting in different serotypes in humans [212, 216, 217]. These observations suggest that antigenically distant vaccines or infections may elicit de novo immunity, by overcoming imprinted immune memory either in the current generation or in immune‐naïve next generations.

## 7 Allelic Preference of Anti‐SARS‐CoV‐2 Antibodies

Many V‐gene segments possess multiple alleles that introduce amino acid variations, thereby enhancing the diversity of the human antibody repertoire within the population. These allelic polymorphisms in immunoglobulin V genes are also found in the CDRs, providing different preferences to antigen recognition. A critical aspect of the antibody response to SARS‐CoV‐2 involves allelic variation in the immunoglobulin genes that encode these antibodies. Recent studies have revealed that specific alleles of heavy and light chains immunoglobulin genes are preferentially utilized during the humoral immune response to SARS‐CoV‐2 infection [61, 218, 219].

**FIGURE 8:**
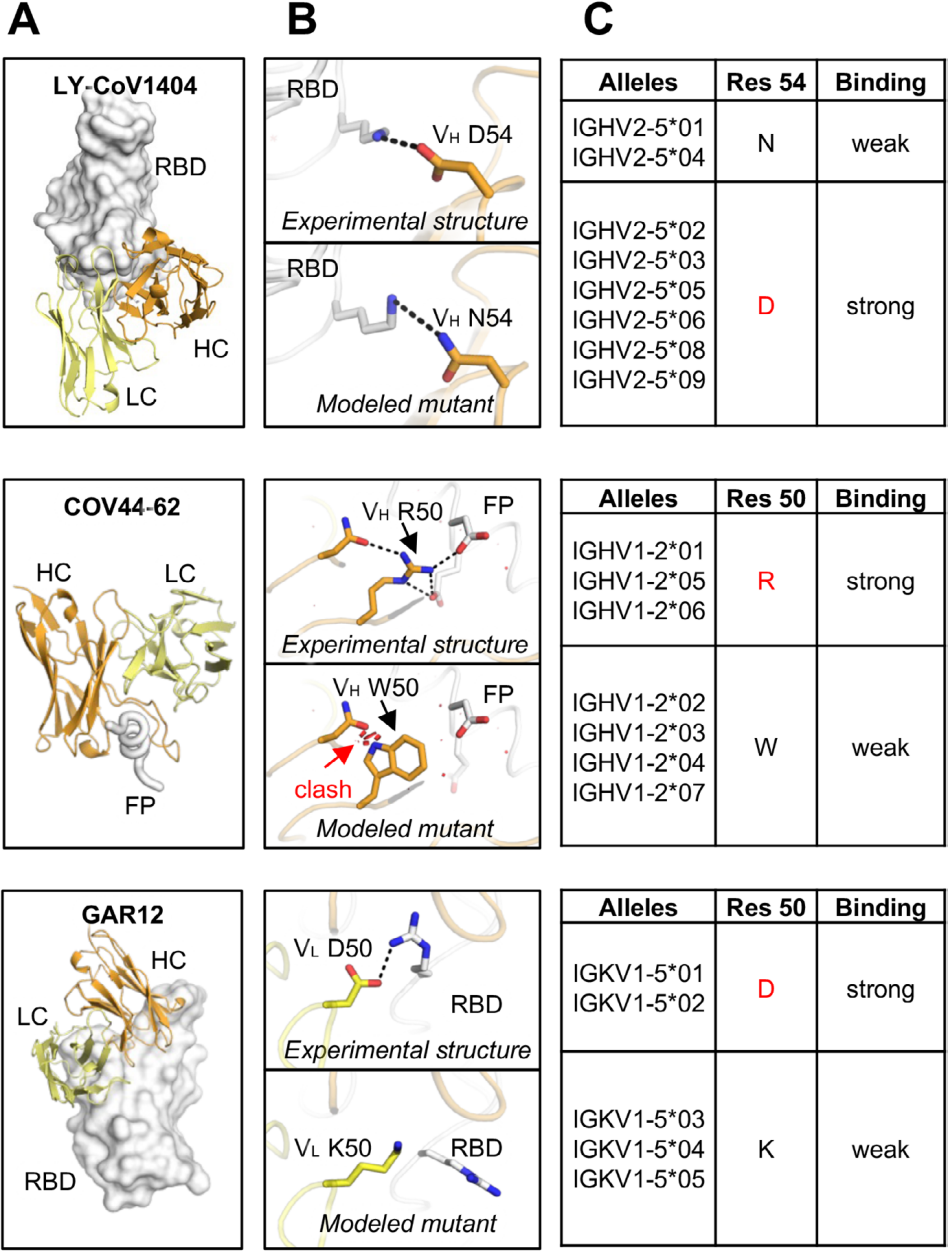
Allelic polymorphisms impact the binding activity of antibodies to SARS‐CoV‐2 antigens. (A) Overall view of antibody/antigen interactions. (B) Detailed interactions between the allelic polymorphic residues and the antigen, as well as the modeled allelic mutations. (C) Allele usage of the paratopic residues. Residues that correspond to the representative antibody in panel A are shown in red. The information of alleles is from the IMGT Repertoire [220]. Binding affinities of the wild‐type and mutated antibodies to SARS‐CoV‐2 S were measured experimentally and reported in our previous studies [61, 218]. Three representative anti‐SARS‐CoV‐2 antibodies with allelic polymorphisms LY‐CoV1404, COV44‐62, and GAR12 are shown here. Other cases are comprehensively analyzed and reported in our previous study [218]. Antibody heavy and light chains are shown in orange and yellow, respectively. Antigens are shown in white. Allelic polymorphic residues are labeled. Red disks indicate significant van der Waals overlap (distance < 2.8 Å), hence representing a steric clash. H bonds and salt bridges are represented by black dashed lines. Kabat numbering is applied to all antibodies. Allelic mutations are modeled by FoldX [221].

IGHV2‐5/IGLV2‐14 encode a public class of antibodies to the RBD that potently cross‐neutralizes a broad range of SARS‐CoV‐2 variants, including omicron and sublineages. Representative antibodies from this class include LY‐CoV1404/bebtelovimab [62], 2–7 [115], S2X324 [65], XGv265 [105], P2S‐2E9 [129], and TH272 [63] (Figure 4). Our sequence analysis indicated a strong allelic preference for IGHV2‐5*02 in these RBD‐targeting antibodies [61]. IGHV2‐5*02 encodes an aspartic acid at position 54, which forms a salt bridge with RBD‐K444, while the alternate allele, IGHV2‐5*01, encodes an asparagine at the same position, which appears to weaken the binding (Figure 8). Mutagenesis analysis confirmed that the D54N mutation reduced RBD binding affinity (*K*_D_) by at least 100‐fold, highlighting the critical role of allelic preference in effective RBD binding [61].

We then extended this analysis to include a comprehensive investigation of antibody allelic polymorphisms in antigen recognition using structures from the Protein Data Bank (PDB) [218]. Through computational and experimental methods, we analyzed over 1000 publicly available antibody–antigen structures and demonstrated that many antibodies contain allelic polymorphisms that can disrupt antibody–antigen interactions. In addition to the IGHV2‐5 polymorphism, we identified other allelic preferences. For instance, the IGHV1‐2*06‐encoded anti‐FP antibody COV44‐62 carries a germline‐encoded V_H_ R50, which provides stronger interactions than the W50 encoded by other alleles (Figure 8). Similarly, the IGKV1‐5*01‐encoded RBD antibody GAR12 uses a V_L_ D50, which forms extensive interactions with the antigen, whereas its counterpart V_L_ K50 disrupts these interactions.

Beyond SARS‐CoV‐2, we demonstrated that this phenomenon is prevalent in antibodies targeting other pathogens, such as influenza A and HIV‐1 [218]. These findings emphasize the importance of genetic diversity in the human immune repertoire and suggest that allelic preferences could be leveraged in the design of therapeutic antibodies.

## 8 Evolutionary Trends of the SARS‐CoV‐2 S Protein Structure

The SARS‐CoV‐2 S protein has undergone significant structural evolution since the onset of the pandemic; mutations have progressively enhanced the virus's ability to infect host cells and evade immune responses. Several key structural trends have emerged during the evolution of the S protein.

### 8.1 Stronger ACE2 Binding Affinity and Antibody Escape

One of the most prominent trends in the evolution of SARS‐CoV‐2 has been the development of mutations that both increase binding affinity to the human ACE2 receptor and enhance antibody escape. Variants have demonstrated progressively stronger ACE2 binding affinities. For instance, the S/ACE2 binding affinity has increased by approximately twofold from the D614G variant to XBB.1.5 [33], and a further two‐ to threefold enhancement has been observed in the BA.2.86 variant [222]. Mutations such as N501Y and S477N have been associated with increased ACE2 binding, contributing to the higher transmissibility of SARS‐CoV‐2 variants [222, 223]. The improved receptor binding has played a key role in the higher transmissibility rates observed in newer variants. Additionally, certain combinations of mutations have shown a synergistic or epistasis effect, further boosting ACE2 binding affinity. For example, the combination of N501Y + Q498R [40] and F456L + Q493E [41, 42] have been identified as leading to enhanced binding affinity. As SARS‐CoV‐2 has evolved, mutations have increasingly enabled the virus to evade host immunity and neutralizing antibodies. In fact, several mutations have been identified as “trade‐off” between antibody escape and host cell binding in the SARS‐CoV‐2 S protein [224]. Overall, the evolutionary trajectory, particularly among Omicron subvariants, has allowed the virus to escape host immunity while maintaining and, in some cases, enhancing viral fitness and transmissibility.

### 8.2 Reoccurring Amino Acids With Other Sarbecoviruses

Many amino acids on SARS‐CoV‐2 S protein are not conserved with other sarbecoviruses. Interestingly, we recent found that SARS‐CoV‐2 evolution has shown a trend of reverting certain amino acids to those found in other sarbecoviruses [225]. For example, the S50L mutation, which has emerged in the SARS‐CoV‐2 variants JN.1 and KP.3, mirrors the leucine conserved across other sarbecoviruses, including SARS‐CoV‐1, RaTG13, WIV1, RsSHC014, and BtKY72. Other mutations observed in major SARS‐CoV‐2 variants—such as F157S, L216F, R403K, F456L, N460K, T478K, V483del, F486P, Q493E, Y505H, P621S, H655Y, and D796Y—are also found in other sarbecoviruses. The recent KP.3.1.1 variant has acquired an additional N‐glycosylation site at N30 through an S31 deletion, a feature also observed in other sarbecoviruses such as pangolin‐CoV GD, pangolin‐CoV GX‐P2V, and RaTG13. Notably, most of these reoccurring mutations have emerged only recently (late 2023 to early 2024), suggesting an accelerated rate of amino acid reoccurrence [225]. This phenomenon points to a convergent evolutionary process that allows SARS‐CoV‐2 to escape immune recognition while retaining or improving its viral fitness. These reoccurring amino acids may play critical roles that are advantageous across the sarbecovirus lineage, offering valuable insights into predicting future SARS‐CoV‐2 variants.

### 8.3 A More Stabilized “RBD‐Down State” in Omicron Subvariants

The S protein can adopt multiple prefusion conformations, with the RBS only accessible when the RBD is in the “up” position, while an RBD in “down” conformation hides the exposure of the RBS. A significant distinction in the omicron BA.1 S protein, compared to earlier SARS‐CoV‐2 variants, is a more favored “down” RBD conformation and more compact structure due to its tighter interprotomer packing [207, 226, 227, 228, 229, 230]. The “3‐down RBD” conformation of prefusion S, which is more favored in omicron subvariants, is potentially an immune‐evasion mechanism by the virus to shield vulnerable regions, such as the CR3022 cryptic site and site V, which would be exposed in an RBD presenting its “up” conformations. However, the balance between evading immune escape by shielding vulnerable regions and hindering ACE2 binding to these same regions is not fully understood.

Over more than 4 years of evolution, the structural changes and mutations in the SARS‐CoV‐2 S protein reveal common trends: the virus is evolving to strengthen receptor binding, escape preexisting antibodies, and evade antibody detection that is related to sometimes stabilizing the “RBD‐down” conformation of the prefusion S protein. Incorporation of recurring amino acid motifs from other sarbecoviruses has also been observed. These structural trends may offer important clues for anticipating the features of future SARS‐CoV‐2 variants.

## 9 Conclusions

In this review, we summarize what is known about where and how antibodies target the S protein of SARS‐CoV‐2 and the current efforts to develop antibodies targeting conserved regions of the SARS‐CoV‐2 spike (S) protein. We have analyzed the structural features of SARS‐CoV‐2 and delineate epitopes where targeted antibodies are evaded by variants, as well as their escape mechanisms. By reviewing antibodies that target different sites, public antibody classes, escape mechanisms, and broadly neutralizing antibodies, we highlight promising directions for structure‐based antibody development against SARS‐CoV‐2 and its variants. While many potent neutralizing antibodies primarily target the variable RBS and are therefore often evaded by viral evolution, antibodies that bind to relatively conserved sites typically do not directly block receptor binding and exhibit modest neutralizing activity. Nonetheless, several exceptional antibodies, such as VYD222, VIR‐7229, SA55, and BD55‐1205, retain high potency against SARS‐CoV‐2 variants (Figure 7). These antibodies highlight two promising sites: VIR‐7229 and BD55‐1205 target RBS‐A, while VYD222 and SA55 target the RBS‐D/CR3022 (class 1/4) site. Notably, two of these four highly escape‐resistant antibodies were generated through in vitro affinity‐maturation strategies, underscoring the potential of enhancing antibodies targeting these sites. Moreover, the remarkable efficacy of BD55‐1205, an antibody from the widely elicited yet often evaded IGHV3‐53/3‐66 public class, suggests the possibility of designing germline‐targeting vaccines aimed at eliciting this antibody lineage.

SARS‐CoV‐2 vaccines are used worldwide and have conferred immune protection against SARS‐CoV‐2 infection. The immune response to COVID‐19 of the majority of the global population is primed and imprinted by the ancestral strain of SARS‐CoV‐2 albeit various vaccine platforms or prior infection. However, as new variants have emerged, the neutralization efficacy of these vaccines has been substantially reduced. The continuously evolving SARS‐CoV‐2 variants, which carry an increasing number of mutations, have been able to escape both humoral and passive immunity. To address this challenge, variant‐based vaccines have been developed and demonstrated improved protection against emerging variants. Booster vaccines containing spike proteins from omicron lineages have been widely administered. In 2022, “bivalent” vaccines targeting both the ancestral SARS‐CoV‐2 strain and omicron subvariants BA.4 and BA.5 were introduced [231]. In August 2024, the US FDA approved and granted EUA for updated mRNA COVID‐19 vaccines (2024–2025 formula) to include a monovalent component that corresponds to the omicron variant KP.2 strain [232]. Due to the rapid evolution of SARS‐CoV‐2, a more universal and broadly effective vaccine against coronaviruses is needed to protect against current and future variants. Furthermore, nearly all FDA‐approved therapeutic antibodies have been rendered ineffective by SARS‐CoV‐2 variants, highlighting the urgent need for developing potent, escape‐resistant antibodies. In the postpandemic era, where SARS‐CoV‐2 continues to evolve and circulate, it is critical to identify epitopes and establish generalizable principles for eliciting escape‐resistant, potent antibodies for therapeutic development and vaccine design. These efforts would lead to pan‐coronavirus vaccines and antibody therapeutics and help protect against a possible SARS‐CoV‐3.

## Conflicts of Interest

The authors declare no conflicts of interest.

## Supporting information


Figure S1.

## Data Availability

The authors have nothing to report.
