# Supplementary material for: Structural Immunology of SARS‐CoV‐2
Source: Immunol Rev. 2024 Dec 27;329(1):e13431. doi: 10.1111/imr.13431 (PMC11727448; doi:10.1111/imr.13431)
Supplement: Supplementary file 1 — Figure S1. [file IMR-329-0-s001.pdf]

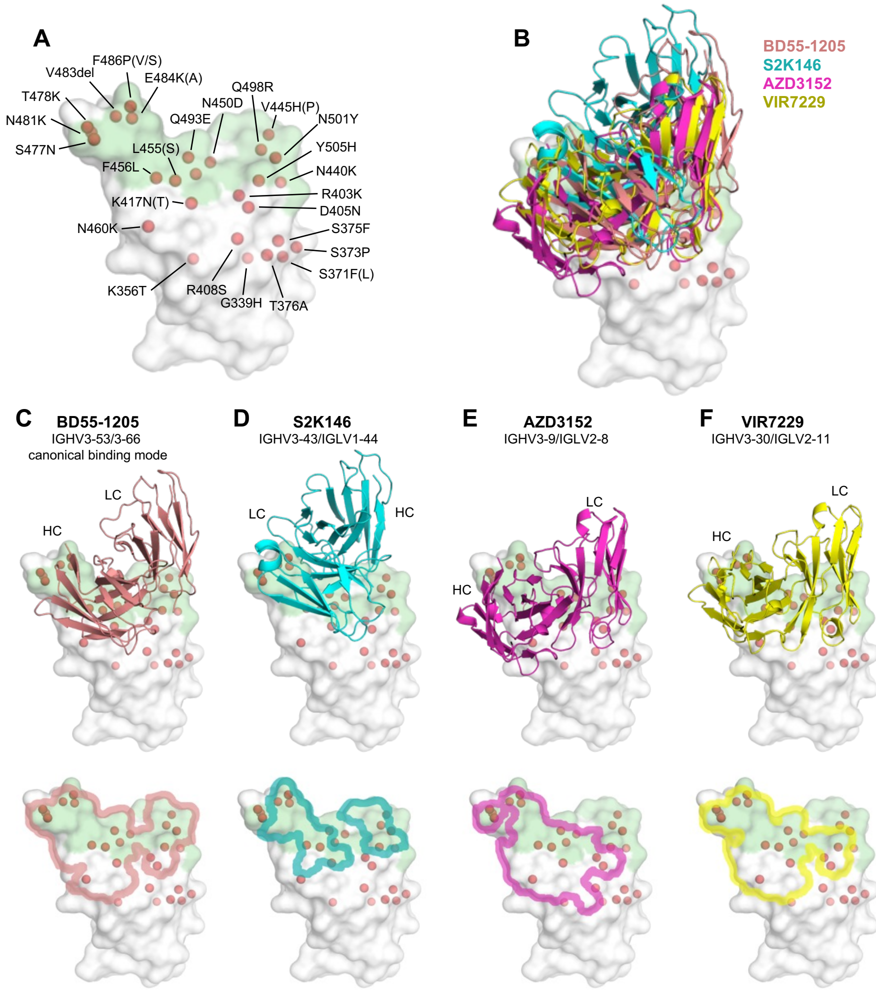

Supplemental Figure 1. Structural comparison of broadly neutralizing antibodies targeting RBS-A. The SARS-CoV-2 RBD is represented by a white surface with the RBS highlighted in light green. Mutated residues of a recent variant KP.3.1.1 compared to the ancestral Wuhan-Hu-1 strain are represented by red spheres. Note that some mutated residues on the other side of the RBD are not shown. **(A)** Highlights of the mutated residues in KP.3.1.1. Alternative mutated residues in non-KP.3.1.1 variants are shown in brackets. **(B)** A superimposition of the variable domains of antibodies BD55-1205 (PDB 8XE9), S2K146 (PDB 7TAS), AZD3152 (PDB 8SUO), and VIR7229 (PDB 9ATM). The antibody/antigen structures are superimposed onto the RBD. **(C-F)** Antibody/antigen structures of the RBS-A-targeting broadly neutralizing antibodies. Epitopes (defined as BSA > 10 Å^2) are outlined in the panels on the bottom.
